# Electron tunneling of hierarchically structured silver nanosatellite particles for highly conductive healable nanocomposites

**DOI:** 10.1038/s41467-020-15709-8

**Published:** 2020-05-07

**Authors:** Daewoo Suh, K. P. Faseela, Wonjoon Kim, Chanyong Park, Jang Gyun Lim, Sungwon Seo, Moon Ki Kim, Hyungpil Moon, Seunghyun Baik

**Affiliations:** 1 School of Mechanical Engineering, Sungkyunkwan University, Suwon, 16419 Republic of Korea; 2 Department of Energy Science, Sungkyunkwan University, Suwon, 16419 Republic of Korea; 3 SKKU Advanced Institute of Nano Technology (SAINT), Sungkyunkwan University, Suwon, 16419 Republic of Korea; 4 Present Address: Production Engineering Research Institute, LG Electronics, Seoul, 07796 Republic of Korea

**Keywords:** Engineering, Materials science, Nanoscience and technology

## Abstract

Healable conductive materials have received considerable attention. However, their practical applications are impeded by low electrical conductivity and irreversible degradation after breaking/healing cycles. Here we report a highly conductive completely reversible electron tunneling-assisted percolation network of silver nanosatellite particles for putty-like moldable and healable nanocomposites. The densely and uniformly distributed silver nanosatellite particles with a bimodal size distribution are generated by the radical and reactive oxygen species-mediated vigorous etching and reduction reaction of silver flakes using tetrahydrofuran peroxide in a silicone rubber matrix. The close work function match between silicone and silver enables electron tunneling between nanosatellite particles, increasing electrical conductivity by ~5 orders of magnitude (1.02×10^3^ Scm^−1^) without coalescence of fillers. This results in ~100% electrical healing efficiency after 1000 breaking/healing cycles and stability under water immersion and 6-month exposure to ambient air. The highly conductive moldable nanocomposite may find applications in improvising and healing electrical parts.

## Abstract (web summary)

Self-healable conductive materials are of importance for emerging electronic technologies. Here, Suh et al. report a nanocomposite exhibiting high conductivity facilitated by electron tunneling between silver nanoparticles and its 100% recovery of conductivity after 1000 breaking and healing cycles.

## Introduction

Healable and deformable conductive materials have received considerable attention for future electronics such as artificial human skin, internet of things, and bioelectronics^1–5^. The healable materials are essential components in robust electronics owing to recoverability after mechanical/electrical damages autonomously or in response to external stimuli^4–7^. A variety of mechanisms, such as metal-coordinated, covalent, and hydrogen bonds, have been investigated to synthesize healable polymer matrix^8–11^. In order to impart electrical conductivity (*σ*), various conductive fillers and conducting polymers were incorporated into the healable polymer matrix^4,6,8–10,12–22^.

The healable conductive nanocomposites can be classified into two types depending on the moldability at room temperature; rigid/flexible/stretchable^4,8–10,12–15^ or moldable viscoelastic nanocomposites^6,16–22^. The rigid/flexible/stretchable nanocomposites with crosslinking exhibited greater elastic behavior with little moldability^4,8–10,12–15^. They provided relatively higher *σ* as shown in Supplementary Table 1. A recent study reported a high *σ* (850 S cm^−1^) with graphene (25 wt%)^10^. The nanocomposite was rigid at room temperature^10^. However, it became bendable and deformable at high temperatures. The excellent healing was achieved by the abundant Zn(II)-carboxylate interactions after heat treatment^10^. In contrast, the moldable nanocomposites showed more viscous behavior and changed shape permanently upon molding, similar to silly putty or playdough^6,16–22^. The elastic property of moldable nanocomposites in literature was typically low to retain their shape after demolding^16–18,20,22^. The moldable nanocomposites provided smaller *σ* than the rigid/flexible/stretchable nanocomposites in literature (Supplementary Table 2). Note that only the healable conductive nanocomposites in literature were listed in Supplementary Tables 1 and 2. Commercially-available conducting playdoughs rely on ionic conduction of electrolytes and possess very low *σ* (≤0.1 S cm^−1^, Supplementary Table 3). Moreover, they show irreversible electrical and mechanical degradation upon drying or heating, limiting practical applications (Supplementary Fig. 1). The incorporation of conductive nanofillers (<2 S cm^−1^) or conducting polymers (≤78 S cm^−1^) still could not provide high conductivity of moldable nanocomposites in previous reports (Supplementary Table 2)^6,16–22^.

About the conducting mechanism, a percolation network with dynamic alignment and physical coalescence of fillers has been suggested as an efficient strategy for typical non-healable nanocomposites^1,23–26^. A high *σ* was achieved by the coalescence of fillers after thermal, optical, or chemical curing^23–26^. However, the coalesced particles were fractured under mechanical deformation or stretching, resulting in an irreversible decrease in *σ*^23–26^. In contrast, non-coalesced percolation networks based on physical contact or electron tunneling of randomly mixed metal/nanocarbon fillers have been investigated for healable nanocomposites^4,8–10,13–22^. However, there was an irreversible degradation in *σ* or mechanical property after the healing process^4,8–10,13–15^. The fillers had to be transported to the damaged area, reoriented, and recontacted, without solid coalescence of fillers, decreasing electrical healing efficiency. The electron tunneling mechanism was also suggested^2,21^, but it was challenging to uniformly disperse nanocarbon fillers within the tunneling distance.

Here we report a highly conductive completely reversible electron tunneling-assisted percolation network of silver nanosatellite (AgNS) particles for putty-like healable and moldable nanocomposites. The hierarchically structured AgNS particles were generated by the unique radical and reactive oxygen species-mediated vigorous etching and reduction reaction of silver flakes (AgFLs) using tetrahydrofuran (THF) peroxide in a healable silicone rubber (SR) matrix. The AgNS network dramatically increased *σ* by ~5 orders of magnitude, achieving an unusually high *σ* (1.02 × 10^3^ S cm^−1^) in putty-like nanocomposites with excellent moldability. The AgNS particles were uniformly and densely distributed with an interparticle distance of 3.1 nm, and the close work function match between Ag and SR enabled electron tunneling. This led to a completely reversible reconstruction of the percolation network, achieving ~100% electrical healing efficiency after 1000 breaking/healing cycles. Moreover, the *σ* was stable even after 1000 water immersion cycles and 6-month exposure to ambient air. An emergency electronics repair demonstration was also carried out by a robot.

## Results

### Synthesis of silver nanosatellite particles

**Fig. 1:**
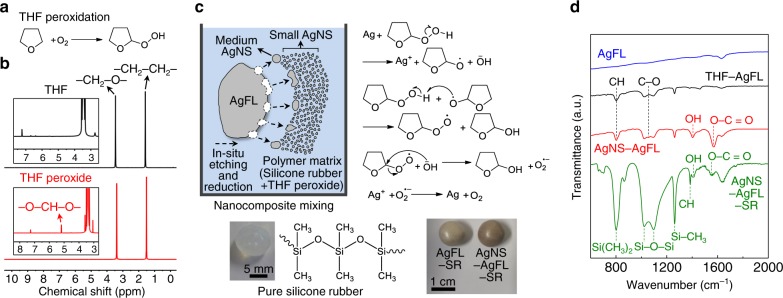
Synthesis of hierarchically structured AgNS particles. **a** Oxygen peroxidizes THF into THF peroxide. **b** ^1^H NMR spectra of THF and THF peroxide. **c** Schematic and chemical mechanism of the radical and reactive oxygen species-mediated vigorous etching and reduction reaction of AgFLs into AgNS particles in a SR matrix. Optical images of the pure SR, AgFL-SR (Ag = 44 vol%), and AgNS-AgFL-SR (Ag = 44 vol%, THF peroxide = 15 mL) nanocomposites are also shown. **d** FTIR spectra of the pure AgFLs, THF-AgFLs, AgNS-AgFLs, and AgNS-AgFL-SR (Ag = 44 vol%, THF peroxide = 15 mL) nanocomposite.

Figure 1 describes synthesis of the AgNS particles by vigorous in-situ etching and reduction reaction of AgFLs (see Methods for details). Firstly, THF was peroxidized by atmospheric oxygen to form THF peroxide (Fig. 1a)^27^. In contrast, the peroxidation could be inhibited by a butylated hydroxytoluene (BHT) inhibitor^28^. Hereafter, the THF containing BHT and peroxidized THF without the inhibitor were referred to as THF and THF peroxide, respectively, unless otherwise specified. The acronyms used in this study are summarized in Supplementary Table 4. The THF peroxidation was confirmed by proton nuclear magnetic resonance (^1^H NMR) analysis (Fig. 1b). A distinct peak was observed at δ = 5.18 ppm for THF peroxide due to oxidation at the α-carbon^29^. However, there was no such peak for THF verifying the absence of peroxidation. The concentration of THF peroxide was 0.068 M (Supplementary Fig. 2 and Supplementary Note 1).

Figure 1c shows the schematic of the AgNS-AgFL-SR nanocomposite. SR was previously employed for putty-like healable nanocomposites due to the hydrogen bonding of cross-linked polydimethylsiloxane chains^18,30^. The vigorous in-situ etching and reduction reaction of microscale AgFLs (~4.3 μm) by THF peroxide in the SR matrix generated hierarchically structured (medium-size and small-size) AgNS particles. The radical and reactive oxygen species-mediated etching and reduction reaction of Ag was proposed as a mechanism, similar to the Ag-hydrogen peroxide (H_2_O_2_) reaction^31–33^. As shown in the reaction schematic of Fig. 1c, THF peroxide etches AgFLs resulting in Ag^+^, THF peroxide radical, and hydroxide anion^31^. The highly reactive THF peroxide radical then forms 2-hydroxy tetrahydrofuran and C_4_H_7_O-OO^•^ radical. In the next step, the reaction between C_4_H_7_O–OO^•^ radical and hydroxide anion generates 2-hydroxy tetrahydrofuran and superoxide anion (O_2_^•−^)^31,32^. Finally, O_2_^•−^ reduces Ag^+^ yielding AgNS particles^33^. Supplementary Fig. 3a and b show optical and scanning electron microscopy (SEM) images of AgFLs treated by THF (THF-AgFLs). AgNS particles were not generated by THF. In contrast, AgNS particles were clearly observed when AgFLs were treated by THF peroxide (AgNS-AgFLs, Supplementary Fig. 3c). AgNS particles were not generated either when the THF peroxide-Ag reaction was carried out with a radical scavenger ((2,2,6,6-Tetramethylpiperidin-1-yl)oxyl), supporting the radical-mediated reaction mechanism (Supplementary Fig. 4). This in-situ etching and reduction reaction was not previously noticed and investigated although AgFLs or graphene were treated by THF in literature^9,34^. Interestingly, there was no oxidation in AgNS-AgFLs (Supplementary Fig. 5). This prevented any decrease in *σ* of nanocomposites as will be discussed later. The generation of AgNS particles changed color of the AgNS-AgFL-SR nanocomposite into dark brown whereas the AgFL-SR nanocomposite (AgFLs treated by THF in SR) was light gray (Fig. 1c inset). The nanocomposite synthesis process, without involving thermal curing, is provided in Supplementary Fig. 6.

Figure 1d compares the Fourier transform infrared (FTIR) spectra of the pure AgFLs, THF-AgFLs, AgNS-AgFLs, and AgNS-AgFL-SR nanocomposite. The CH and C–O peaks related with THF were observed for the THF-AgFLs. An additional strong O–C=O peak (1569 cm^−1^), corresponding to conjugated γ-butyrolactone, was observed for the AgNS-AgFLs. The 2-hydroxy tetrahydrofuran, formed in the reaction medium, generated conjugated γ-butyrolactone as a byproduct upon further oxidation^35,36^. Additional peaks related with SR (Si(CH_3_)_2_ stretching, Si–O–Si stretching, Si–CH_3_ stretching, and CH bending) were observed for the AgNS-AgFL-SR nanocomposite^37^. Note that there was no chemical reaction between THF peroxide and SR itself (Supplementary Fig. 7).

The peroxidation process was further investigated by the NMR analysis. The ^1^H NMR analysis with dimethyl sulfone as an internal standard indicated that the concentration of THF peroxide was 0.061 M (Supplementary Fig. 8a and Supplementary Note 2). This was close to the iodometric titration result (Supplementary Fig. 2, 0.068 M). The ^13^C NMR analysis also confirmed the existence of peroxidation. The THF with BHT inhibitor exhibited two peaks (Supplementary Fig. 8b). In contrast, the oxidation at the α-carbon atom of THF exhibited four distinct peaks of THF peroxide (Supplementary Fig. 8c). The ^1^H NMR analysis was also carried out after the reaction of THF peroxide with AgFLs (AgNS-AgFL, Supplementary Fig. 8d). The residual THF peroxide peak was negligible after the AgNS particle generating reaction.

### Characterization of silver nanosatellite particles

**Fig. 2:**
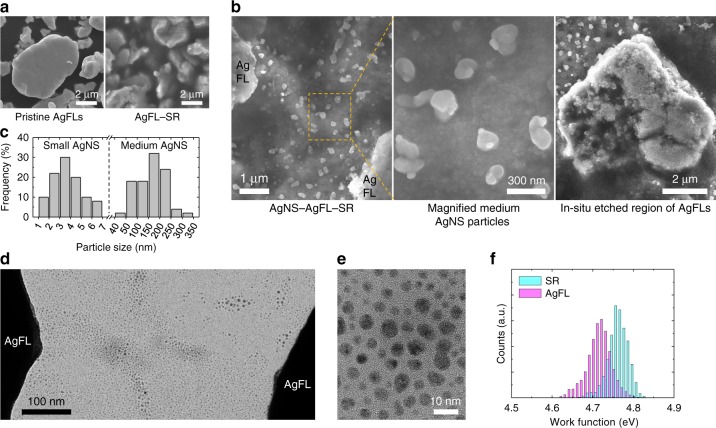
Characterization of medium and small AgNS particles. **a, b** SEM images of the AgFL-SR (Ag = 44 vol%, THF = 15 mL) and AgNS-AgFL-SR (Ag = 44 vol%, THF peroxide = 15 mL) nanocomposites without heating. **c** Size distribution of small and medium AgNS particles. The sum of each frequency is 100%. **d, e** TEM images of small AgNS particles (THF peroxide-treated AgFLs). The polymer was excluded to get clear images. **f** Work function distribution of pure SR and AgFLs.

There was no AgNS particle generation in the AgFL-SR nanocomposite synthesized using the THF with BHT inhibitor (Fig. 2a). The SEM image of pristine AgFLs is also shown in Fig. 2a. The smooth edges of AgFLs were clearly observed. In contrast, AgNS particles were generated when AgFLs were treated by THF peroxide in SR matrix at room temperature, due to the radical and reactive oxygen species-mediated vigorous etching and reduction process (as discussed in Fig. 1c). The unique hierarchically structured AgNS particles with a bimodal size distribution were categorized as the medium and small AgNS particles. Figure 2b (left and middle) show the in-situ generated medium AgNS particles between microscale AgFLs. The average particle size of the medium AgNS particles was 164 nm (Fig. 2c). Figure 2b (right) shows rough edges and dimples of the AgFL where medium AgNS particles were etched from. The severely etched AgFL surface could also be observed when the THF peroxide amount was increased to 45 mL (Supplementary Fig. 9). The area fraction of medium AgNS particles was 10.5% (Supplementary Fig. 10). Figure 2d, e show transmission electron microscopy (TEM) images of densely and uniformly distributed small AgNS particles. The average size of small AgNS particles was 3.7 nm with an interparticle distance of 3.1 nm (Fig. 2c). There was no small AgNS particle generation when AgFLs were treated using the THF with BHT inhibitor (Supplementary Fig. 11).

The in-situ formation of AgNPs from AgFLs was also previously reported^2,12^. Ag ions were diffused from the Ag_2_O layer of AgFLs embedded in the fluorine rubber matrix and reduced into AgNPs (average size: 8.1 nm) by the fluorine surfactant and thermal curing process (80 and 120 °C, 1 hr each)^2^. The diffusion continued with time, increasing the size of AgNPs and forming nanorods^2^. The possibility of exfoliation of AgNPs from AgFLs was excluded^2^. Another work also reported the in-situ formation of AgNPs by diffusion of Ag ions from AgFLs^12^. The diffused ions were reduced into AgNPs by the carbonyl groups of self-healing polymer matrix^12^. In contrast, we do not rely on the diffusion mechanism to synthesize the medium and small AgNS particles. The reduction was achieved without the aid of surfactant/polymer and thermal curing process to generate AgNS particles (Supplementary Fig. 3c). Both the medium and small AgNS particles were synthesized by the unique radical and reactive oxygen species-mediated vigorous etching and reduction reaction with THF peroxide (Fig. 1c). THF peroxide with radical scavenger or THF with BHT inhibitor could not generate AgNS particles (Supplementary Figs. 4 and 11). There was no change in electrical conductivity of the AgNS-AgFL-SR nanocomposites after 6-month storage in an ambient air environment, excluding the diffusion mechanism (as will be discussed in Supplementary Fig. 23a). The unique AgNS particles with bimodal (medium and small) size distribution enabled nearly perfect healing efficiency (~100%) as will be discussed later. The stretching cycle durability and/or healing efficiency were limited in previous reports, without additional encapsulation layer, increasing resistance with repeated cycles^2,12^. This work was also different from our previous report where the solvated Ag ions were directly mixed with the polymer solution^38^. There was no Ag ion diffusion or etching process^38^. The solvated Ag ions were then thermally reduced into AgNPs^38^.

The electron tunneling has been previously suggested as a transport mechanism between nanoparticles in nanocomposites^2,21^. The electron tunneling depends on the height (*V*) and width (*d*) of potential barrier, which is described by the Simmons approximation^39^.

1 $${\it{I}} \propto {\mathrm{exp}}\left( {\frac{{ - 2{\it{d}}\sqrt {2{\it{m}}^ \ast {\it{V}}} }}{{\it{h}}}} \right)$$

where *I* is the electron tunneling current, m* is the effective mass of electron, and *h* is the Planck’s constant^39^. The *V* is determined from the work function difference between AgFLs and SR matrix. Figure 2f shows the work function distributions of SR and AgFLs measured by Kelvin probe force microscopy (KPFM). The mean work function of AgFLs (4.71 eV) was consistent with the previous report measured by ultraviolet photoelectron spectroscopy^40^. The close work function match between Ag and SR lowered the barrier height and enabled electron tunneling between small AgNS particles with an interparticle distance of 3.1 nm.

### Electrical transport property of the nanocomposites

**Fig. 3:**
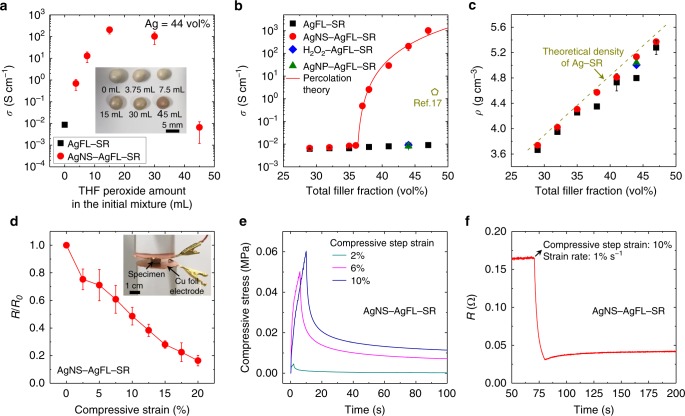
Electrical transport property of the AgNS-AgFL-SR nanocomposite. **a** The electrical conductivity of the AgNS-AgFL-SR nanocomposite as a function of THF peroxide amount in the initial mixture. The error bars represent the standard deviation of the data. The Ag filler fraction was fixed at 44 vol%. Optical images of the nanocomposites are provided in the inset. **b**, **c** The electrical conductivity and density of the AgNS-AgFL-SR nanocomposites are compared with those of control specimens as a function of the total Ag filler fraction. The density was measured by the Archimedes method. The amount of THF and THF peroxide was fixed at 15 mL. The electrical conductivity of the graphite-incorporated healable viscoelastic nanocomposite is also compared^17^. The error bars represent the standard deviation of the data. **d** The normalized resistance of the AgNS-AgFL-SR (Ag = 44 vol%, THF peroxide = 15 mL) nanocomposite as a function of compressive strain. The error bars represent the standard deviation of the data. The resistance was measured in steady state after relaxation at each strain. The inset image shows the measurement setup. **e, f** The mechanical stress and electrical resistance relaxation of the AgNS-AgFL-SR (Ag = 44 vol%, THF peroxide = 15 mL) nanocomposite after compressive step strain (strain rate = 1% s^−1^).

The electrical transport property of the AgNS-AgFL-SR nanocomposite is shown in Fig. 3. The *σ* was measured using a square prism-shaped specimen (Supplementary Fig. 12). Figure 3a shows the *σ* of the AgNS-AgFL-SR nanocomposite (Ag = 44 vol%) as a function of THF peroxide amount in the initial mixture. The *σ* of the AgFL-SR specimen, synthesized without THF peroxide, was only 8.64 × 10^−3^ S cm^−1^. Surprisingly, the in-situ etching and reduction reaction of AgFLs by the optimum THF peroxide amount (15 mL) increased *σ* by ~5 orders of magnitude, achieving 2.06 × 10^2^ S cm^−1^. This was striking because a highly conductive electrical percolation network was constructed only by electron tunneling of AgNS particles, bridging AgFL islands, without curing-induced physical coalescence of fillers. The carrier mobility and concentration of the optimum AgNS-AgFL-SR specimen, measured by the van der Pauw method, were 1.59 cm^2^ V^−1^ s^−1^ and 1.69 × 10^21^ cm^−3^, respectively. The reproducibility of the AgNS-AgFL-SR nanocomposite was good over multiple batches (Supplementary Fig. 13). The *σ* decreased beyond the optimum THF peroxide amount due to the excessive etching of AgFLs. The dimples and defects on the severely-etched AgFLs acted as electron scattering sites (Supplementary Fig. 9). The density (*ρ*) of the AgNS-AgFL-SR nanocomposite was also maximum at the optimum THF peroxide amount (Supplementary Fig. 14).

**Fig. 4:**
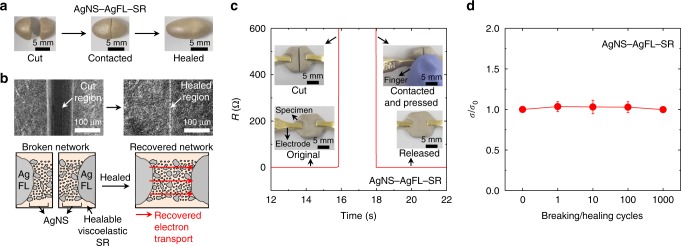
Healable electrical transport of the AgNS-AgFL-SR nanocomposite. **a, b** Optical and SEM images of the AgNS-AgFL-SR (Ag = 44 vol%, THF peroxide = 15 mL) nanocomposite after breaking and healing. Schematics of the healable AgNS network are also provided. **c** The resistance change of the AgNS-AgFL-SR nanocomposite during a breaking/healing cycle. An optical image at each step is also provided. **d** The normalized electrical conductivity of the AgNS-AgFL-SR nanocomposite is shown as a function of the breaking/healing cycles. The error bars represent the standard deviation of the data.

**Fig. 5:**
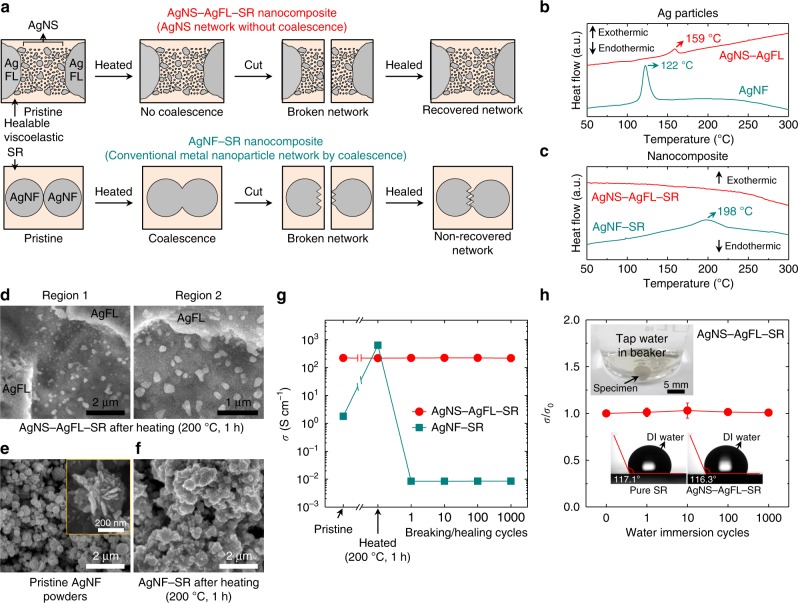
Electrical network healing mechanism of the AgNS-AgFL-SR nanocomposite. **a** Schematic of the healing mechanism. Tunneling-assisted, non-coalesced, hierarchically structured AgNS particle network between AgFLs is shown above. Conventional electrical network construction by metal nanoparticle coalescence is shown below. **b** DSC analysis of AgNS-AgFLs and AgNFs without the SR matrix. **c** DSC analysis of AgNS-AgFL-SR (Ag = 44 vol%, THF peroxide = 15 mL) and AgNF-SR (AgNF = 20 vol%) nanocomposites. **d** SEM images of two different regions of the AgNS-AgFL-SR nanocomposite after the heating process (200 °C, 1 h). **e, f** SEM images of the uncured pristine AgNF powders and cured AgNF-SR nanocomposite (200 °C, 1 h, AgNF = 20 vol%). A magnified SEM image of a pristine AgNF is provided in the inset. **g** The electrical conductivity of the AgNS-AgFL-SR and AgNF-SR nanocomposites before and after heating (200 °C, 1 h) and subsequent breaking/healing cycles. **h** The normalized electrical conductivity of the AgNS-AgFL-SR nanocomposite as a function of water immersion cycles. The error bars represent the standard deviation of the data. An optical image of the water immersion process (1 min for each cycle) and water contact angles on the pure SR and AgNS-AgFL-SR specimens are also provided. The conductivity was measured after removing residual water on the specimen.

**Fig. 6:**
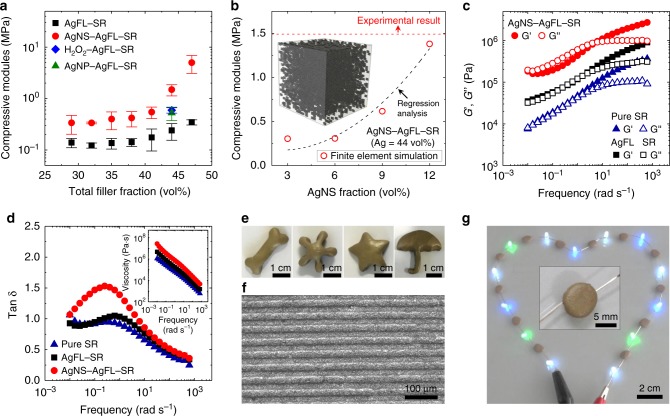
Mechanical properties and moldability of the AgNS-AgFL-SR nanocomposite. **a** Compressive modulus of the healable nanocomposites. The error bars represent the standard deviation of the data. **b** The finite element analysis of the AgNS-AgFL-SR nanocomposite as a function of the AgNS particle fraction. The total Ag filler fraction was fixed at 44 vol%. A numerical model (AgNS = 3 vol%) is provided in the inset. The experimentally measured compressive modulus of the AgNS-AgFL-SR (Ag = 44 vol%) nanocomposite is designated by a dashed line. **c** Storage (*G*’) and loss (*G*”) moduli of the pure SR, AgFL-SR, and AgNS-AgFL-SR nanocomposites as a function of shear strain frequency (shear strain amplitude = 1%, 25 °C). **d** The tan δ of the pure SR, AgFL-SR, and AgNS-AgFL-SR (Ag = 44 vol%, THF peroxide = 15 mL) nanocomposites. The dynamic viscosity is provided in the inset. **e**, **f** Macroscale and microscale (width: 20 μm, spacing: 20 μm, height: 10 μm) moldability of the AgNS-AgFL-SR nanocomposite. **g** Light emitting diode circuit employing random-shaped AgNS-AgFL-SR interconnectors. A magnified optical image is provided in the inset.

**Fig. 7:**
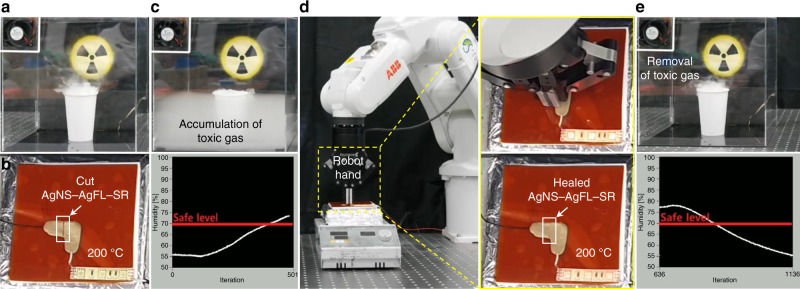
The robot application demonstration of the AgNS-AgFL-SR nanocomposite. **a** An exhaust fan was connected to the healable AgNS-AgFL-SR (Ag = 44 vol%, THF peroxide = 15 mL) nanocomposite circuit at 200 °C, removing toxic gas at harsh conditions. **b, c** The circuit was cut, accumulating the toxic gas and increasing the gas concentration (relative humidity) above the safe level. **d** The robot healed the AgNS-AgFL-SR circuit. The healed circuit image is provided in the inset. **e** The fan restarted, removing the toxic gas and decreasing the gas concentration below the safe level.

Figure 3b shows the *σ* of the AgNS-AgFL-SR nanocomposite as a function of the Ag filler fraction (THF peroxide = 15 mL). Note that the in-situ etching and reduction reaction of AgFLs and generation of AgNS particles did not change the total Ag fraction. Surprisingly, there was no enhancement in *σ* of the AgFL-SR nanocomposite, synthesized with THF, even when the Ag fraction increased up to 47 vol% (9.07 × 10^−3^ S cm^−1^). The *σ* could be increased to 1.35 × 10^−1^ S cm^−1^ (AgFL = 47 vol%) only after an additional thermal curing process (200 °C, 1 h). In contrast, the *σ* of the AgNS-AgFL-SR nanocomposite increased dramatically, even without the thermal curing process, when the Ag fraction was greater than 36 vol%. The electron tunneling-assisted AgNS percolation network showed a good agreement with the 3D percolation theory.

2 $$\sigma = \sigma _0\left( {{\it{V}}_{\mathrm{f}} - {\it{V}}_{\mathrm{c}}} \right)^s$$

where *σ*_0_ is the electrical conductivity of Ag, *V*_f_ is the filler volume fraction, *V*_c_ is the experimentally obtained percolation threshold (0.36), and *s* is the fitting exponent^23^. The maximum *σ* of the AgNS-AgFL-SR nanocomposite was 1.02 × 10^3^ S cm^−1^ (Ag = 47 vol%). The mechanical healability and moldability started to degrade at excessive Ag filler fractions, and the optimized Ag concentration was selected as 44 vol%. The AgNS-AgFL-SR nanocomposite synthesized at the optimized condition (Ag = 44 vol%, THF peroxide = 15 mL) was primarily investigated in Figs. 3–7, unless otherwise specified.

As a control, a H_2_O_2_-AgFL-SR nanocomposite (Ag = 44 vol%) was synthesized by mixing H_2_O_2_-treated AgFLs with SR. The concentration of H_2_O_2_ was the same as that of the THF peroxide (0.068 M). There was only a slight increase in *σ* (9.13 × 10^−3^ S cm^−1^) compared with the AgFL-SR nanocomposite. The cyclic voltammetry analysis of AgFLs was carried out using the THF peroxide or H_2_O_2_ electrolyte (Supplementary Fig. 15). The oxidation peak was observed at a higher bias potential for the H_2_O_2_. This demonstrated that AgFLs were more resistant to oxidation in the H_2_O_2_ environment, generating a smaller number of AgNS particles. The H_2_O_2_ etching generated only a small number of AgNPs (<20 nm) even at a higher concentration (0.68 M, Supplementary Fig. 16). This demonstrated the efficient reaction of THF peroxide, generating densely and uniformly distributed AgNS particles. As another control, commercial AgNPs (~293 nm, 10.5 vol%) were mixed with AgFLs (33.5 vol%) in SR matrix using the THF with inhibitor instead of THF peroxide (AgNP-AgFL-SR nanocomposite). The size and concentration of AgNPs were similar to those of medium AgNS particles (Supplementary Fig. 17a). The *σ* of the AgNP-AgFL-SR nanocomposite (8.29 × 10^−3^ S cm^−1^) was significantly smaller than that of the AgNS-AgFL-SR nanocomposite (2.06 × 10^2^ S cm^−1^). The commercial AgNPs with a smaller diameter (~30 nm, 10.5 vol%) were also investigated (Supplementary Fig. 17b). The *σ* of the AgNP-AgFL-SR nanocomposite (7.83 × 10^−3^ S cm^−1^, AgNP = ~30 nm) was similar to that of the nanocomposite with bigger AgNPs (8.29 × 10^−3^ S cm^−1^, AgNP = ~293 nm). The commercial AgNPs could not mimic the in-situ generated AgNS particles which had both medium (~164 nm) and small (~3.7 nm) sizes. This demonstrated the unique role of the in-situ AgNS particles synthesized by THF peroxide, which could not be achieved by other methods.

The *σ* of the AgNS-AgFL-SR nanocomposite was also compared with those of putty-like or playdough-like healable and moldable nanocomposites in literature (Fig. 3b). The literature data, where the filler volume fraction or *σ* information is not available, are summarized in Supplementary Table 2. The maximum *σ* of the moldable conductive filler-polymer matrix nanocomposites was only 1.98 S cm^−1^^17^. One moldable nanocomposite made of conducting polymer provided 78 S cm^−1^^6^. However, the concept of filler fraction was not applicable to the conducting polymer since the conduction mechanism was completely different. The maximum *σ* of the AgNS-AgFL-SR nanocomposite (1.02 × 10^3^ S cm^−1^) was about 3 orders of magnitude greater than that of the moldable conductive filler-polymer matrix nanocomposites in literature^17^. As shown in Fig. 3c, the *ρ* of AgNS-AgFL-SR nanocomposite was close to the theoretical value calculated by the rule of mixture^41^. The densely and uniformly distributed fillers were embedded in the polymer matrix without air voids. The other control specimens showed a lower *ρ* probably due to the air voids which scattered electrons and decreased *σ*.

As shown in Fig. 3d, the electrical resistance (*R*) of the AgNS-AgFL-SR nanocomposite decreased monotonically with compression primarily due to the geometry change. The steady state *R* was recorded after initial relaxation period at each strain. The dynamic stress relaxation behavior upon compressive step strain was also investigated (Fig. 3e). The stress rapidly increased upon step strain followed by a slow (40–50 s) relaxation behavior. The large stress relaxation was consistent with the characteristics of viscous moldable materials^6^. There was also a rapid decrease in *R* upon step strain followed by a slow relaxation (Fig. 3f). The electrical relaxation duration (40–50 s) was similar to the stress relaxation duration. This indicated that small AgNS particles immediately rearranged and reconstructed tunneling-assisted percolation network, during the stress relaxation period, stabilizing electrical transport property. Note that randomly mixed graphene fillers in a viscoelastic polymer matrix exhibited a much slower electrical relaxation than stress relaxation possibly due to the diffusion, reorientation, and recontact of fillers^18^.

### Healable electrical transport and healing mechanism

Figure 4 shows the mechanical and electrical healing process of the AgNS-AgFL-SR nanocomposite. Healibility typically stems from the dynamic chemical bonds of polymers^11,30^. The healibility of SR is attributed to the hydrogen bonding as it contains both hydrogen bond donor (oxygen atom in dimethylsiloxane network) and acceptor (hydrogen atom)^30^. The bifurcated AgNS-AgFL-SR nanocomposites were immediately healed by a gentle finger touch for ~2 s (Fig. 4a and b). The applied pressure was 194 kPa (Supplementary Fig. 18). Figure 4c shows the change in *R* of the AgNS-AgFL-SR nanocomposite during a breaking/healing cycle. The *R* increased to infinity after bifurcation of the specimen. Surprisingly, it immediately recovered to its initial value (~0.1 Ω) upon gentle touch of the bifurcated specimens. The AgNS particles were already densely and uniformly distributed in the SR matrix within the electron tunneling distance. These particles immediately reconstructed the percolation network upon touch (Fig. 4b schematic), without the need for diffusion, re-orientation, and recontact of fillers. The *σ* of the AgNS-AgFL-SR nanocomposite was completely recovered (electrical healing efficiency, *σ*/*σ*_0_ = ~100%) even after 1000 breaking/healing cycles (Fig. 4d). The specimen was reshaped into a square prism after the breaking/healing cycles for the precise *σ* measurement by the four-point probe in-line method (Supplementary Fig. 12). The previously reported healable nanocomposites could not provide perfect electrical healing efficiency (Supplementary Tables 1 and 2). This demonstrated the excellence of the AgNS particles for the repeatable electrical percolation network construction for healable nanocomposites.

The compressive modulus (*E*) of the AgNS-AgFL-SR nanocomposite was almost invariant during 20 breaking/healing cycles, indicating reversible mechanical property (Supplementary Fig. 19). The specimen was compressed, manually bifurcated, healed, and reshaped for each stress-strain experiment. The tensile stress-strain characteristics were also investigated during 20 breaking/healing cycles (Supplementary Fig. 20). The dumbbell-shaped AgNS-AgFL-SR (Ag = 47 vol%, THF peroxide = 15 mL) specimen was clamped, stretched, bifurcated, healed, and reshaped for each tensile stress-strain experiment. Almost completely reversible mechanical properties were observed, realizing ~100% healing efficiency in tensile modulus, although there was somewhat fluctuation. The fluctuation in the data could be due to the slightly different shape of the dumbbell-shaped test specimen for each tensile test, because it was manually formed using a polylactic acid mold. There was no degradation in mechanical properties after the repeated tensile tests.

The electrical transport property of the AgNS-AgFL-SR nanocomposite was also investigated after thermal curing (200 °C, 1 h) in order to further elucidate the nature of the AgNS percolation network (Fig. 5a). Differential scanning calorimetry (DSC) analysis was employed to investigate coalescence behavior of the AgNS particles at high temperatures (Fig. 5b and c). There was an exothermic peak at 159 °C for the AgNS-AgFL particles, indicating coalescence of Ag particles (Fig. 5b)^41,42^. However, the exothermic peak disappeared when the AgNS particles were embedded in the SR matrix (Fig. 5c). The AgNS particles were uniformly encapsulated by SR, preventing coalescence up to 300 °C. The coalescence of AgNS particles could not be observed from SEM images even after the heating process (200 °C, 1 h) as shown in Fig. 5d. Resultantly, there was no increase in *σ* of the AgNS-AgFL-SR nanocomposite even after the thermal curing process (Fig. 5g). The electrical transport was still realized by the tunneling-assisted percolation network of AgNS particles. This was advantageous for reconstruction of the percolation network, maintaining high and stable *σ* for healable nanocomposites, since there was no fracture of the coalesced fillers during the breaking process (Fig. 5a). Indeed, there was no change in *σ* of the thermally-cured AgNS-AgFL-SR nanocomposite during 1000 breaking/healing cycles. The AgNS-AgFL-SR nanocomposite started to decompose after 400 °C (Supplementary Fig. 21). The cyclic temperature-sweep rheology measurement of the AgNS-AgFL-SR nanocomposite is also carried out (Supplementary Fig. 22). The storage (*G*’) and loss (*G*”) moduli return to the initial values at room temperature when the maximum temperature is 80 °C. The variation in *G*’ and *G*” is less than an order of magnitude in the investigated temperature range although there is somewhat hysteresis. However, there is an increase in *G*’ and *G*” at room temperature when the maximum temperature is higher (100 and 120 °C). This could be due to the evaporation of the remnant solvent. Nevertheless, the electrical conductivity could be healed after the curing at 200 °C for 1 h as discussed in Fig. 5g.

As a control, flower-shaped Ag nanoparticles (AgNFs) were mixed with SR matrix (Fig. 5a, AgNF-SR nanocomposite). AgNFs had a hierarchical nanostructure with 2-dimensional thin petals (~12 nm) radially protruded out of a spherical bud (~400 nm) (Fig. 5e)^24,41,43^. The DSC analysis revealed a strong exothermic peak at 122 °C for AgNFs, indicating active coalescence of thin petals (Fig. 5b)^41,43^. Unlike the AgNS-AgFL-SR nanocomposite, AgNFs embedded in SR still exhibited an exothermic coalescence peak at 198 °C (Fig. 5c) although the sintering temperature increased due to the wrapping polymer. The coalesced AgNFs could be clearly observed (Fig. 5f). The randomly-mixed AgNFs were coalesced due to the imperfect encapsulation by the polymer matrix. Consequently, the *σ* of the AgNF-SR nanocomposite significantly increased from 1.80 to 632 S cm^−1^ after the thermal curing process (200 °C, 1 h, Fig. 5g). The *σ* of the typical filler-polymer matrix nanocomposites in literature also increased after thermal curing process^2,23,38,43^. However, the *σ* dramatically decreased to 8.48 × 10^−3^ S cm^−1^ after the first breaking/healing cycle due to the fracture of coalesced AgNFs (Fig. 5a and g). The *σ* could not be recovered during the 1000 breaking/healing cycles.

Strikingly, there was no change in *σ* of the AgNS-AgFL-SR nanocomposite during 1000 water immersion cycles due to the hydrophobic nature of the SR (Fig. 5h). The *σ* and *E* were also stable for 6 months in an ambient air environment (Supplementary Fig. 23). The AgNS-AgFL-SR nanocomposite maintained stability under harsh conditions which is useful for practical industrial applications.

### Mechanical property, moldability, and application

Figure 6a compares the *E* of the nanocomposites as a function of the Ag filler fraction. The *E* was estimated from stress-strain curves (Supplementary Fig. 24). The AgNS-AgFL-SR nanocomposite exhibited greater *E* than the AgFL-SR nanocomposite in the entire filler fraction. The *E* of the AgNS-AgFL-SR nanocomposite was also greater than those of the H_2_O_2_-AgFL-SR and AgNP-AgFL-SR nanocomposites (Ag = 44 vol%). As shown in Fig. 6b, a finite element analysis (FEA)^44^ was carried out in order to compare the *E* of the nanocomposite before and after the in-situ etching and reduction process (see Supplementary Note 3 for details). The AgNS-AgFL-SR nanocomposite was modeled as a unit cube (side length = 0.4 µm) and considered to be composed of two parts (Fig. 6b inset). Before the in-situ etching process, the nanocomposite was composed of the polymer matrix and Ag flakes (AgFL-SR nanocomposite) which was taken as a reference matrix of the model. The stress–strain characteristics of this reference matrix were directly obtained from the experimental measurements of the AgFL-SR nanocomposite (Fig. 6a). The AgNS particles, modeled as cubic elements with a similar size (4 nm), constituted the second part. The effect of the AgNS particles on the *E* of the AgNS-AgFL-SR nanocomposite was simulated by FEA. This approach allowed the direct comparison of the simulation results with the experimental data (Fig. 6a). Four numerical models with different AgNS fractions (3, 6, 9, 12 vol%) were created (Supplementary Fig. 25). The simulated *E* of the AgNS-AgFL-SR nanocomposite increased as the AgNS fraction increased (Fig. 6b), which was consistent with the experimental data (Fig. 6a). The AgNS fraction was found to be 12.2 vol%, from the regression analysis (goodness of fit = 93.1%), to match the experimentally measured *E*. The stress distribution at 1% compressive strain is provided in Supplementary Fig. 26. A larger stress was generated around the AgNS particles since the stiffer AgNS particles experienced a much heavier load, compared with the reference matrix. Furthermore, the reference matrix exhibited local stiffening in the AgNS particle rich region as if the particles were connected. This was reasonable because the reference matrix had a smaller *E* than the AgNS particles, absorbing the impact of compression.

The *G*’ and *G*” were also measured as a function of frequency (Fig. 6c). The *G*’ and *G*” of the pure SR were similar in the low frequency range (<6.3 rad s^−1^), demonstrating a viscoelastic behavior. The addition of AgFLs to SR (AgFL-SR) increased both *G*’ and *G*”. The in-situ etching and reduction reaction of AgFLs (AgNS-AgFL-SR) further increased *G*’ and *G*”. Interestingly, the relative magnitude of *G*” became greater than *G*’ in the low frequency range. This was also observed in the damping factor (tan δ = *G*”/*G*’, Fig. 6d)^1,6,45^. The tan δ of the AgNS-AgFL-SR nanocomposite was greater than one in the low frequency range (<6.3 rad s^−1^). The densely and uniformly dispersed AgNS particles made the nanocomposite more viscous. This is favorable for energy dissipation from mechanical shock or vibration^1,45^. The addition of AgFLs and AgNS particles increased the dynamic viscosity (*η*) (Fig. 6d inset). The *η* decreased with increasing shear strain rate, demonstrating a shear thinning behavior. This indicated that there was no severe aggregation or alignment of the AgNS particles under high-rate mechanical deformation^17^. Figure 6e and f show excellent macroscale and microscale moldability of the AgNS-AgFL-SR nanocomposite. Various different shapes were easily formed by the simple molding process.

As an application demonstration, the highly conductive AgNS-AgFL-SR nanocomposite was employed as random-shaped electrical interconnectors, stably operating light-emitting diodes (Fig. 6g). An emergency electronics repair demonstration was also performed by a robot using the AgNS-AgFL-SR nanocomposite (Fig. 7 and Supplementary Movie 1). This is useful for accidents at places where humans cannot enter, due to the leakage of toxic gas and high temperature. An exhaust fan was connected to the healable nanocomposite circuit, which was heated to 200 °C, removing toxic gas at harsh conditions (Fig. 7a). A situation was simulated where the circuit was cut, accumulating the toxic gas and increasing the gas concentration above the safe level (Fig. 7b and c). The dry ice in water was used as a simulant for the toxic gas, and relative humidity was monitored. A robot was dispatched to the emergency site, healing the AgNS-AgFL-SR circuit (Fig. 7d). The fan restarted, removing the toxic gas and decreasing the gas concentration below the safe level (Fig. 7e).

## Discussion

The vigorous in-situ etching and reduction reaction of microscale AgFLs by THF peroxide generated uniformly distributed medium (164 nm) and small (3.7 nm) AgNS particles in a healable and moldable SR matrix (AgNS-AgFL-SR nanocomposite) at room temperature. The close work function match between Ag and SR enabled electron tunneling between small AgNS particles (interparticle distance = 3.1 nm). This constructed electron tunneling-assisted percolation network between AgFL islands without physical coalescence of fillers. This increased *σ* by ~5 orders of magnitude, achieving an extraordinary high *σ* (1.02 × 10^3^ S cm^−1^) for putty-like nanocomposites. The ~100% healing efficiency was maintained even after 1000 breaking/healing cycles since the percolation network relied on electron tunneling rather than physical coalescence of fillers. The *σ* was also stable under harsh conditions such as 1000 water immersion cycles and 6-month exposure to ambient air. The AgNS particles increased *G”* in the low frequency range, resulting in excellent moldability with shear thinning behavior. An emergency electronics repair demonstration was also performed by a robot. The highly conductive, moldable, healable, and stable AgNS-AgFL-SR nanocomposite is promising for future electronic materials.

## Methods

### Synthesis of moldable nanocomposites

The AgNS-AgFL-SR nanocomposite was synthesized by the following process. THF without inhibitor (Sigma-Aldrich, 401757) was exposed to ambient air for a week to prepare THF peroxide (0.068 M). THF with butylated hydroxytoluene inhibitor (Sigma-Aldrich, 186562) was also employed to prevent THF peroxidation. The viscoelastic SR (Wacker Chemie, Elastosil R 401/10, 15 wt%) was dissolved in the THF with inhibitor by stirring for 24 h at room temperature to prepare a polymer matrix solution. AgFLs (Metalor, SA-31812, 29–47 vol%) were dispersed in the SR matrix solution (2 g) with additional THF peroxide (0–45 mL) by tip sonication (Sonictopia, STH-750S, 525 W, 10 min) and subsequent stirring to generate AgNS particles. The mixture was then drop-casted on a Teflon petri dish and dried overnight to obtain the AgNS-AgFL-SR nanocomposite.

The AgFL-SR (AgFL = 29–47 vol%) nanocomposite was synthesized by dispersing AgFLs in the SR matrix solution (2 g) with additional THF with inhibitor (15 mL), instead of THF peroxide. The AgNP-AgFL-SR nanocomposite was synthesized by dispersing both commercial AgNPs (Inframat Advanced Materials, 47MN-06, ~293 nm, 10.5 vol% or CNVISION, Ag Nanopowder, ~30 nm, 10.5 vol%) and AgFLs (33.5 vol%) in the SR matrix solution (2 g) with additional THF with inhibitor (15 mL). AgNFs (0.735 g, 20 vol%), synthesized following a previously published protocol^43^, were mixed with the SR matrix solution (2 g) with additional THF with inhibitor (15 mL) to synthesize the AgNF-SR nanocomposite.

The H_2_O_2_-AgFL-SR nanocomposite was also prepared. Firstly, H_2_O_2_ (Sigma-Aldrich, 216763, 9.77 M) was diluted using ethanol to obtain an H_2_O_2_ solution (0.068 or 0.68 M, 15 mL). In the next step, AgFLs (2.311 g) were treated by the H_2_O_2_ solution and dried overnight to obtain H_2_O_2_-AgFL powders. The H_2_O_2_-AgFL powders were then mixed with the SR matrix solution (2 g) with additional THF with inhibitor (15 mL). The ratio of the AgFLs (2.311 g) to H_2_O_2_ (15 mL) was the same as that of the AgNS-AgFL-SR (Ag = 2.311 g at 44 vol%, THF peroxide = 15 mL, 0.068 M) nanocomposite.

### Characterization

The *σ* was measured by the four-point probe in-line method using a laboratory-built device^23^. The mean values and standard deviations of multiple specimens (≥3) were evaluated at each condition in Figs. 3–5. The *R* was measured by the two-point probe method using a direct current power supply (Keithley, 2280S-32-6). The resistance of the probing wires and contact electrodes was subtracted from the total resistance to precisely measure the resistance of specimens^38^. The work function was measured by KPFM (Nanonavi, E-sweep) using a rhodium-coated silicone probe (Nanoworld, SI-DF3-R). The measurement was calibrated using a highly oriented pyrolytic graphite. The *ρ* was measured by the Archimedes method (Sartorius, Quintix224-1SKR). The NMR (Bruker, AVANCEIII700, 700 MHz) analysis was carried out using CDCl_3_ (δ = 7.26 ppm, Sigma-Aldrich, 151858). The specimens were analyzed by FTIR (Bruker, IFS-66/S, TENSOR27), SEM (Jeol, JSM-7500F), TEM (Jeol, JEM-2100F), XRD (Smart Lab, Cu Kα radiation at 1.5418 Å, 45 kV, and 200 mA), XPS (Thermo Scientific, ESCALAB250), DSC (Seiko, DSC7020, 25–300 °C, 10 °C min^−1^, nitrogen atmosphere), and TGA (Seiko, TG/DTA7300, 25–800 °C, 10 °C min^−1^, nitrogen atmosphere). The water contact angles were measured (SmartDrop, FEMTOFAB, SDL200TEZD). The stress-strain characteristics were measured by a universal testing machine (Instron, 3343). The rheological property was measured by a rheometer (TA Instruments, ARES-G2). The cyclic voltammetry analysis was also carried out (CH Instruments, Electrochemical Workstation, CHI660C).

### Emergency electronics repair by a robot

A robot arm (ABB, IRB120) equipped with a robot hand (ROBOTIQ, 2F-85) performed the emergency electronics repair using the AgNS-AgFL-SR nanocomposite. The robot was controlled using a robot controller (ABB, IRC5) and a teach pendant. The fog effect was created using the dry ice (Taekyung Chemical) in water. The relative humidity was also monitored (Grove-THO2 sensor).

## Supplementary information

Supplementary Information

Description of Additional Supplementary Files

Supplementary Movie 1

## Data Availability

The authors declare that the main data supporting the findings of this study are available within the article and its Supplementary Information files. All other relevant data are available from the corresponding author upon reasonable request.

## Code availability

The code used to calculate the results for this work is available from the authors upon reasonable request.
