# Supplementary material for: Electron tunneling of hierarchically structured silver nanosatellite particles for highly conductive healable nanocomposites
Source: Nat Commun. 2020 May 7;11:2252. doi: 10.1038/s41467-020-15709-8 (PMC7206115; doi:10.1038/s41467-020-15709-8)
Supplement: Supplementary file 1 — Supplementary Information [file 41467_2020_15709_MOESM1_ESM.pdf]

Supplementary Information

Electron tunneling of hierarchically structured silver nanosatellite particles for highly conductive healable nanocomposites

Daewoo Suh et al.

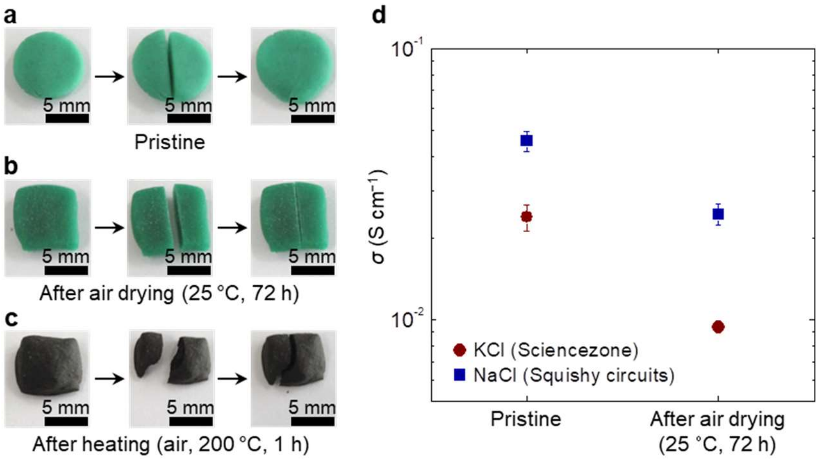

Supplementary Figure 1 Healability of commercial conductive playdoughs. a–c Optical images of playdough specimens (Squishy circuits) before and after air drying (25 °C, 72 h) or heating (200 °C, 1 h). The fresh specimen was healable by one-time finger-pressing (~194 kPa). However, it was not healable after air drying (25 °C, 72 h) or heating (200 °C, 1 h). **d** Electrical conductivity of playdough specimens (Sciencezone and Squishy circuits) before and after air drying (25 °C, 72 h). The error bars represent the standard deviation of the data.

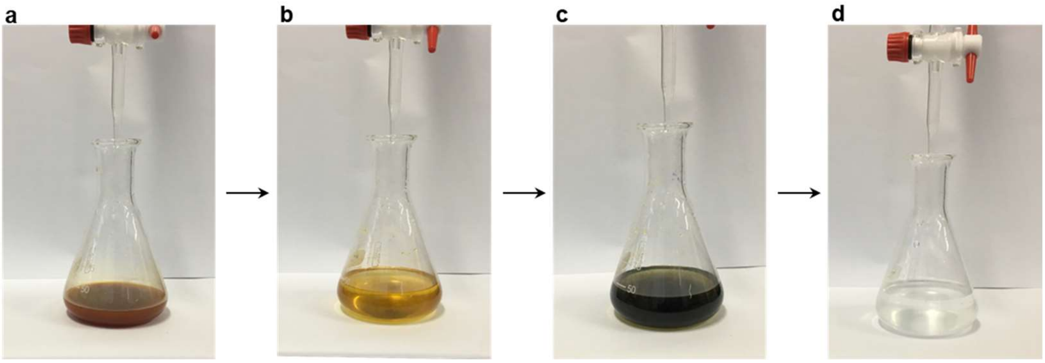

Supplementary Figure 2 Iodometric titration of THF peroxide. **a** Analyte mixture solution. **b** The mixture solution after adding $Na_2S_2O_3$. **c** The mixture solution after adding starch. **d** The mixture solution at the end of titration.

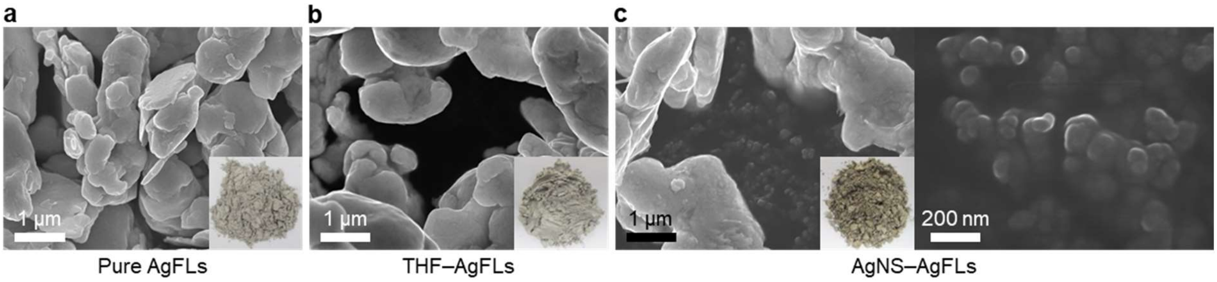

Supplementary Figure 3 SEM and optical images (inset) of AgFLs. The polymer matrix was excluded to obtain clear images, and the thermal curing process was not employed. **a** Pristine AgFLs. The color of the pristine AgFLs was light gray as shown in the inset optical image. **b** THF-AgFLs (THF with BHT inhibitor-treated AgFLs, THF with BHT inhibitor = 15 mL). There was no color change in the THF-AgFLs (inset). **c** AgNS-AgFLs (THF peroxide-treated AgFLs, THF peroxide = 15 mL). The color of the powder changed from light gray to light brown (inset) indicating the formation of AgNS particles [1].

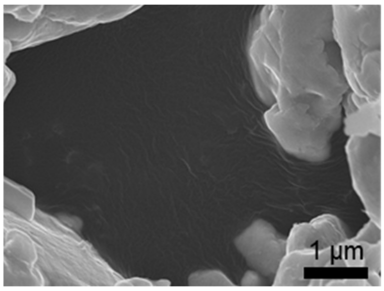

Supplementary Figure 4 SEM image of the THF peroxide-treated AgFLs. The reaction was carried out with the radical scavenger ((2,2,6,6-Tetramethylpiperidin-1-yl)oxyl (TEMPO), 0.2 M in THF peroxide).

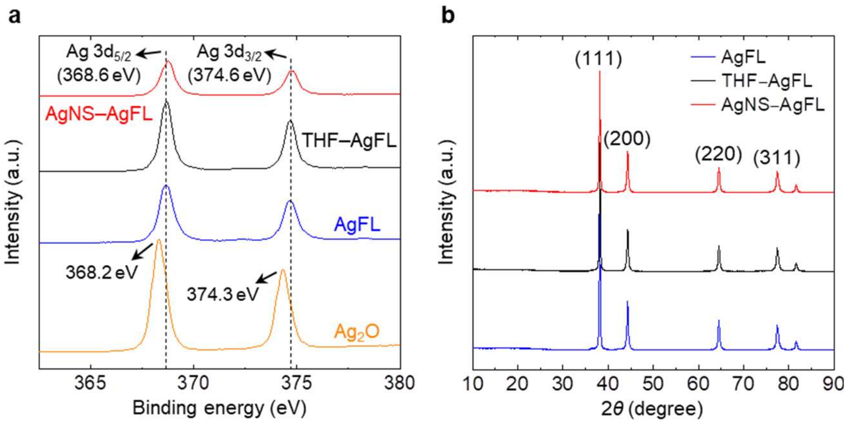

Supplementary Figure 5 X-ray spectroscopy analysis. a X-ray photoelectron spectroscopy (XPS) of the pristine AgFLs, THF-AgFLs, and AgNS-AgFLs. The experimentally measured XPS data of commercial silver oxide particles (Ag_2O, Alfa Aesar, 42577) are also provided to compare the oxidation status of Ag particles. **b** X-ray diffraction analysis.

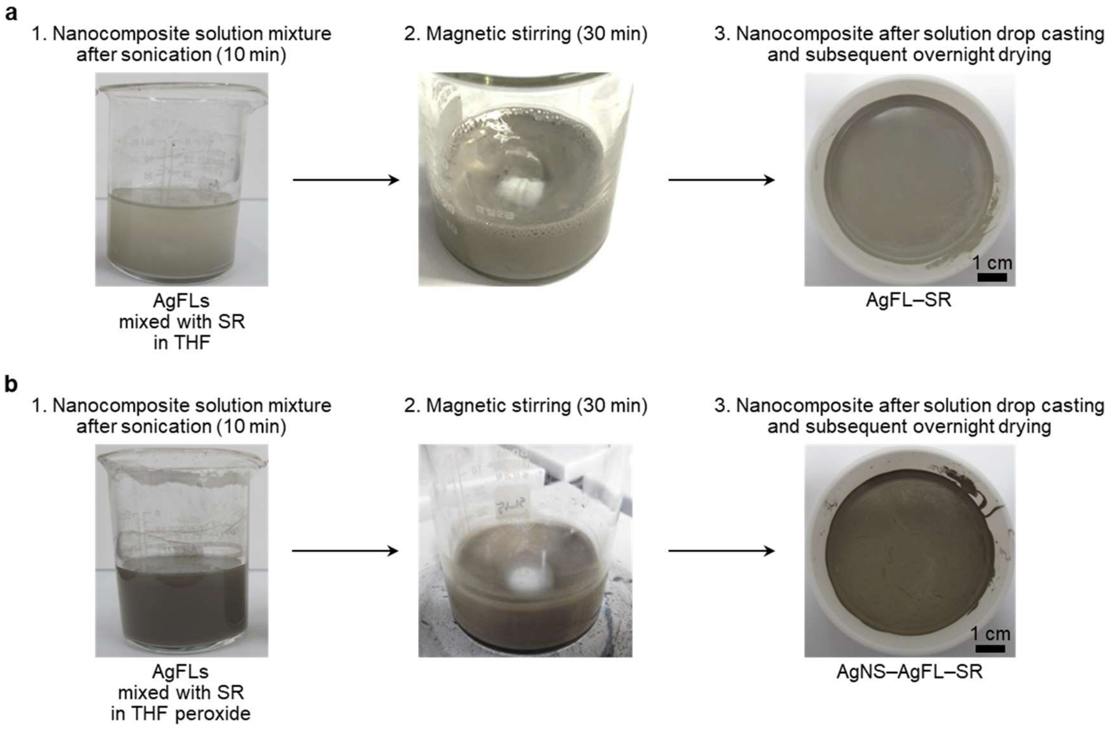

Supplementary Figure 6 Synthesis process of the nanocomposites. a AgFL-SR nanocomposite **b** AgNS-AgFL-SR nanocomposite.

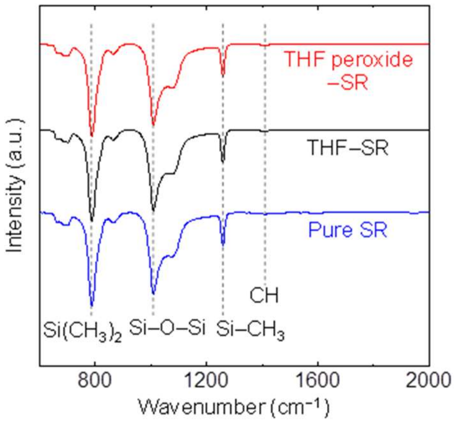

Supplementary Figure 7 Fourier transform infrared spectroscopy analysis. The pure SR, THF-SR (THF-treated SR), and THF peroxide-SR (THF peroxide-treated SR) specimens were compared. The peaks corresponding to the SR matrix were $Si(CH_3)_2$ stretching, Si-O-Si stretching, Si-CH_3 stretching, and CH bending. There was no chemical reaction of SR with THF or THF peroxide.

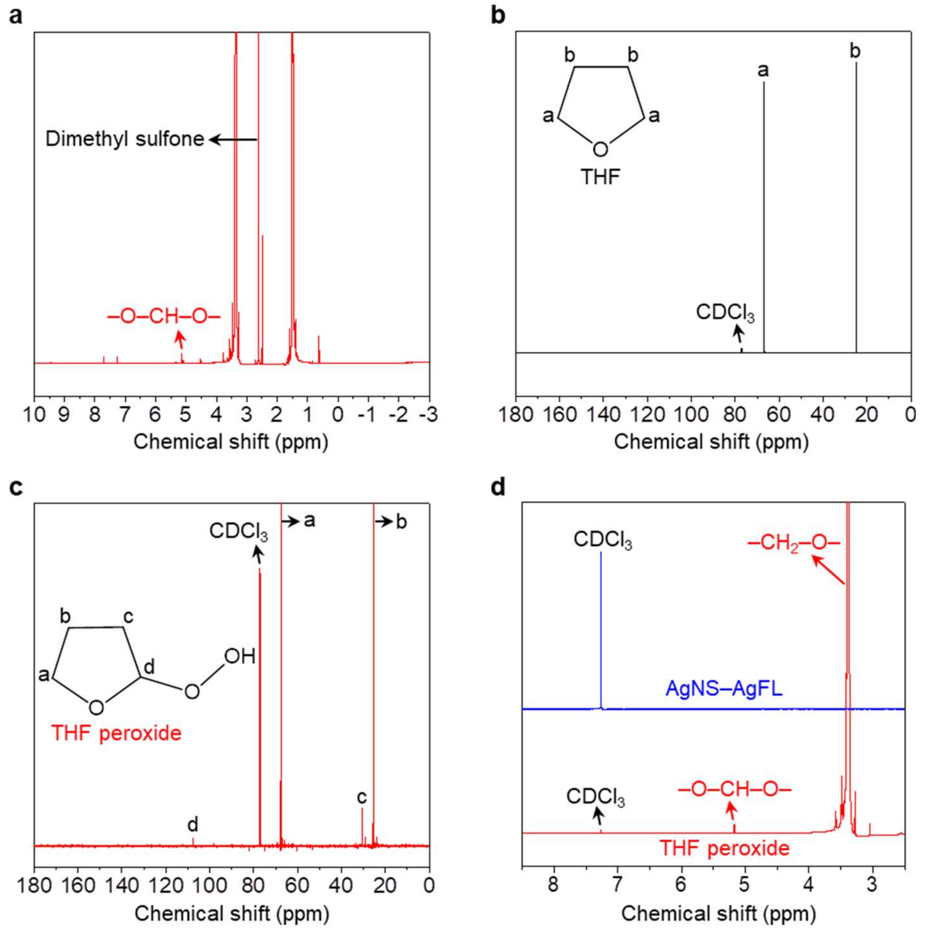

Supplementary Figure 8 The NMR analysis of the peroxidation process. a ^{1}H NMR spectrum of THF peroxide containing dimethyl sulfone (0.154 M) as an internal standard. **b, c** ^{13}C NMR analysis of THF with BHT inhibitor and THF peroxide. The oxidation at the α-carbon atom of THF changed the electronic environment, resulting in four distinct peaks of THF peroxide. **d** ^{1}H NMR analysis of THF peroxide-treated Ag flakes (AgNS-AgFL) and pure THF peroxide. The THF peroxide peak was negligible for AgNS-AgFL.

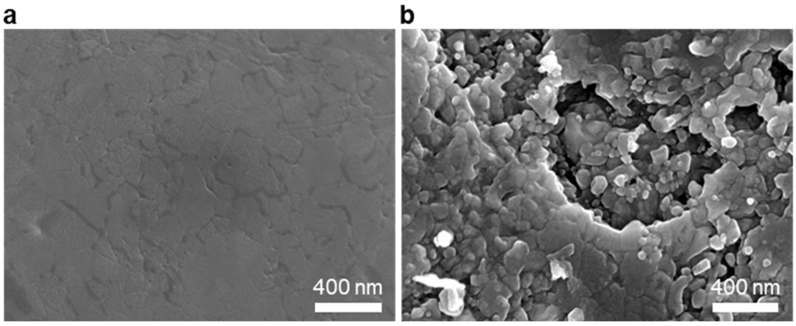

Supplementary Figure 9 Magnified surface SEM images. **a** Pristine AgFL. **b** THF peroxide-treated AgFL (THF peroxide = 45 mL). The polymer matrix was excluded to get clear images.

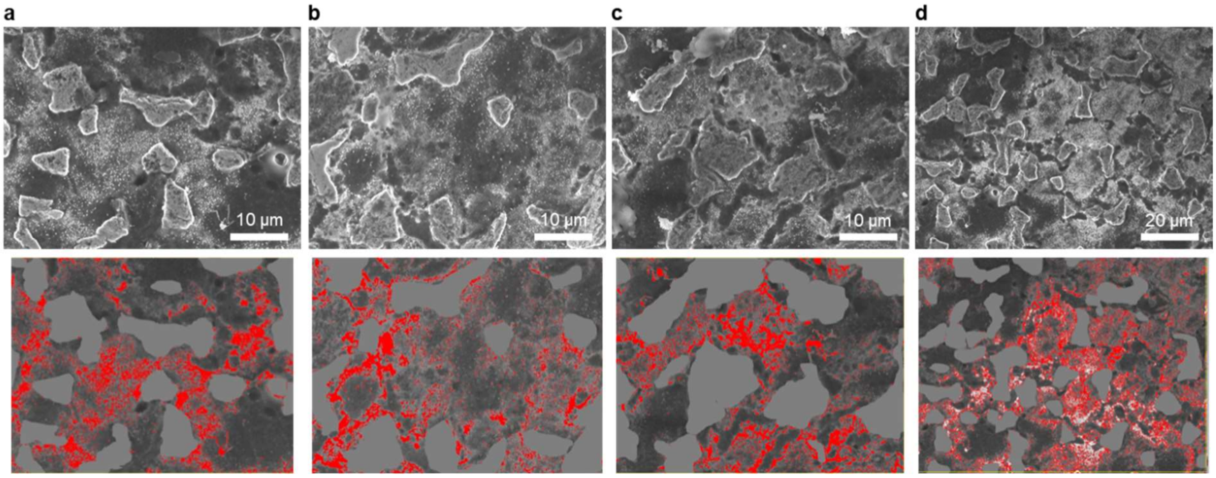

Supplementary Figure 10 The SEM area fraction analysis. **a-d** The average area fraction of the medium AgNS particles in the AgNS-AgFL-SR (Ag = 44 vol%, THF peroxide = 15 mL) nanocomposite was 10.5% (ImageJ software).

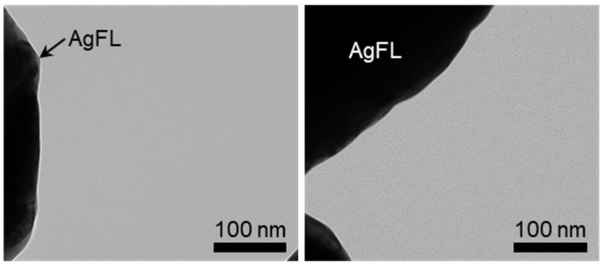

Supplementary Figure 11 TEM image analysis. The two different regions of the THF-AgFLs (THF with BHT inhibitor-treated AgFLs) are shown.

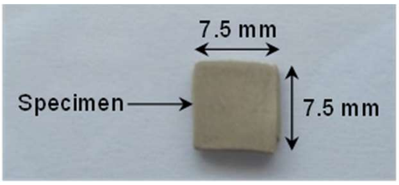

Supplementary Figure 12 An optical image of the nanocomposite. The AgNS-AgFL-SR (Ag = 44 vol%, THF peroxide = 15 mL) nanocomposite (7.5 × 7.5 × 1.0 mm^3) was used for the electrical conductivity (σ) measurement. The σ was measured by the four-point probe in-line method using a current source (Keithley 6221) and a nanovoltmeter (Keithley 2182A) [2]. The distance between tungsten probes was 1 mm. The σ was calculated by the following equation.

$$\sigma = \frac{I}{GV}$$

where I is the supplied current, V is the measured voltage, and G is the geometric factor of the specimen. A detailed description about the geometry calibration is provided elsewhere [2, 3].

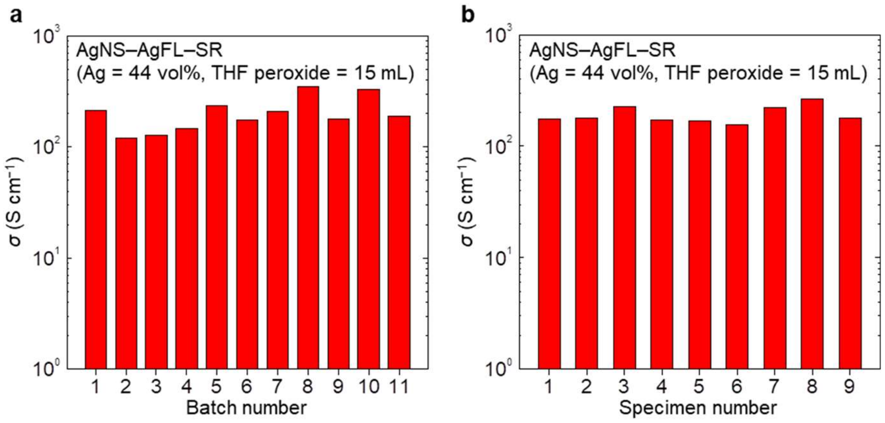

Supplementary Figure 13 The electrical conductivity of the AgNS-AgFL-SR nanocomposites. a The data from multiple batches. **b** The data from multiple specimens from the same batch.

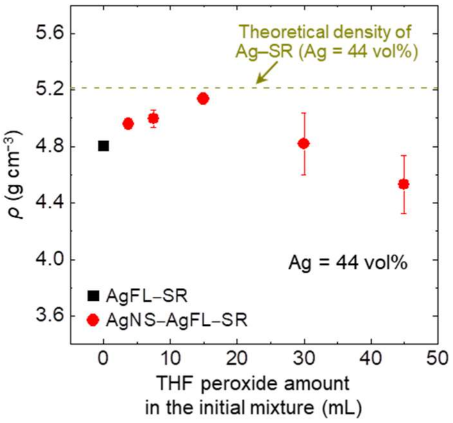

Supplementary Figure 14 The density of the AgNS-AgFL-SR nanocomposite. The density was measured by the Archimedes method as a function of the THF peroxide amount in the initial mixture. The error bars represent the standard deviation of the data.

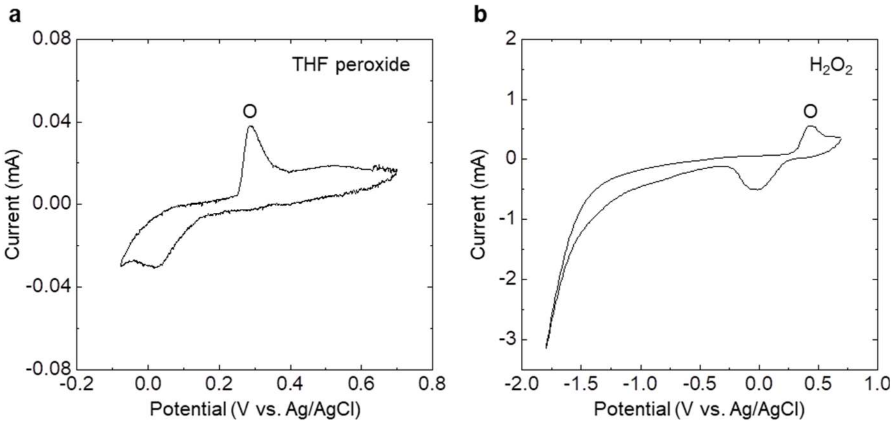

Supplementary Figure 15 The cyclic voltammetry analysis. a THF peroxide electrolyte. **b** H_2O_2 electrolyte. The scan rate was 10 mV s^{-1}. The concentration of the THF peroxide and H_2O_2 was identical (0.068 M). The AgFLs deposited on a glassy carbon electrode, Ag/AgCl electrode, and platinum wire were used as the working, reference, and counter electrodes, respectively. The oxidation peak was marked using a circular symbol. The oxidation peak was observed at a higher bias potential (0.44 V) for the H_2O_2 electrolyte, compared with that (0.29 V) for the THF peroxide electrolyte.

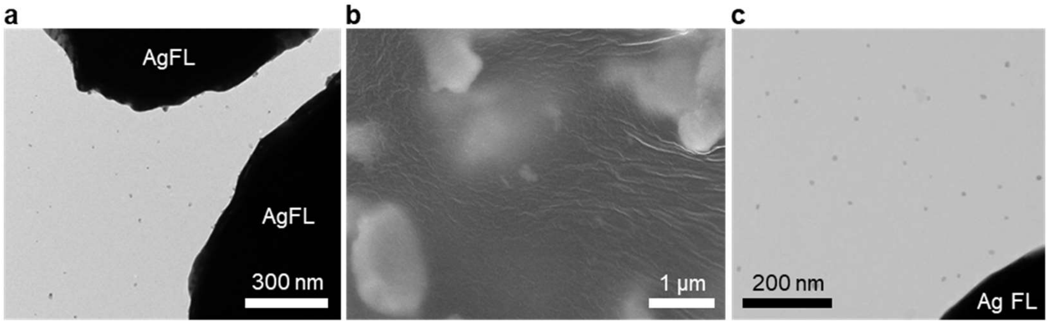

Supplementary Figure 16 H_2O_2-treated AgFLs. a TEM image of the H_2O_2-AgFLs (H_2O_2 = 0.068 M). **b** SEM image of the H_2O_2-AgFL-SR (Ag = 44 vol%, H_2O_2 = 0.068 M) nanocomposite. **c** TEM image of the H_2O_2-AgFLs (H_2O_2 = 0.68 M).

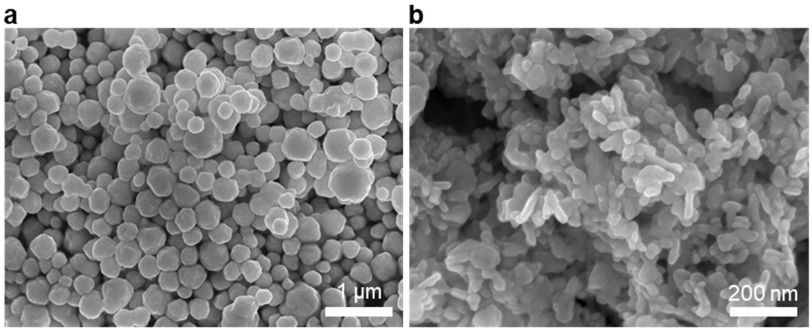

Supplementary Figure 17 SEM images of commercially obtained AgNPs. a AgNPs with an average diameter of ~293 nm. **b** AgNPs with an average diameter of ~30 nm.

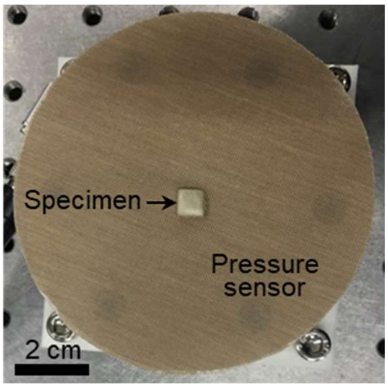

Supplementary Figure 18 Pressure measurement set-up for the healing finger touch. The AgNS-AgFL-SR (Ag = 44 vol%, THF peroxide = 15 mL) nanocomposite specimen is also shown.

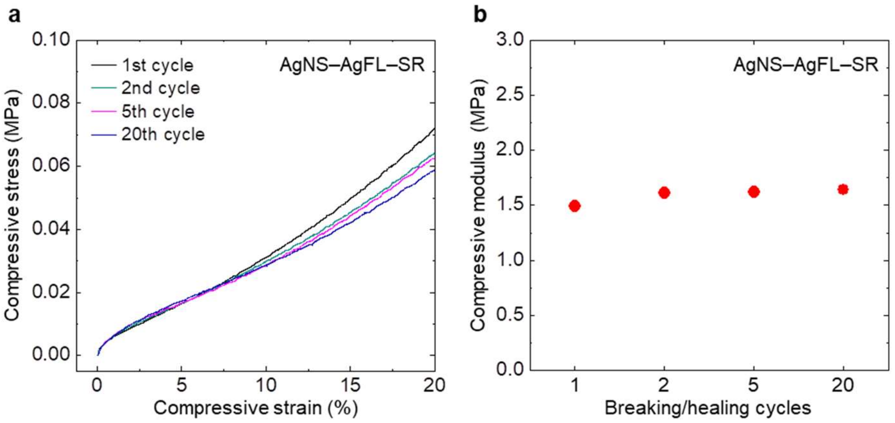

Supplementary Figure 19 The compressive stress-strain characteristics. a The stress-strain data of the AgNS-AgFL-SR (Ag = 44 vol%, THF peroxide = 15 mL) nanocomposite during 20 breaking/healing cycles. The specimen was compressed, manually bifurcated, healed, and reshaped for each stress-strain experiment. **b** Compressive modulus.

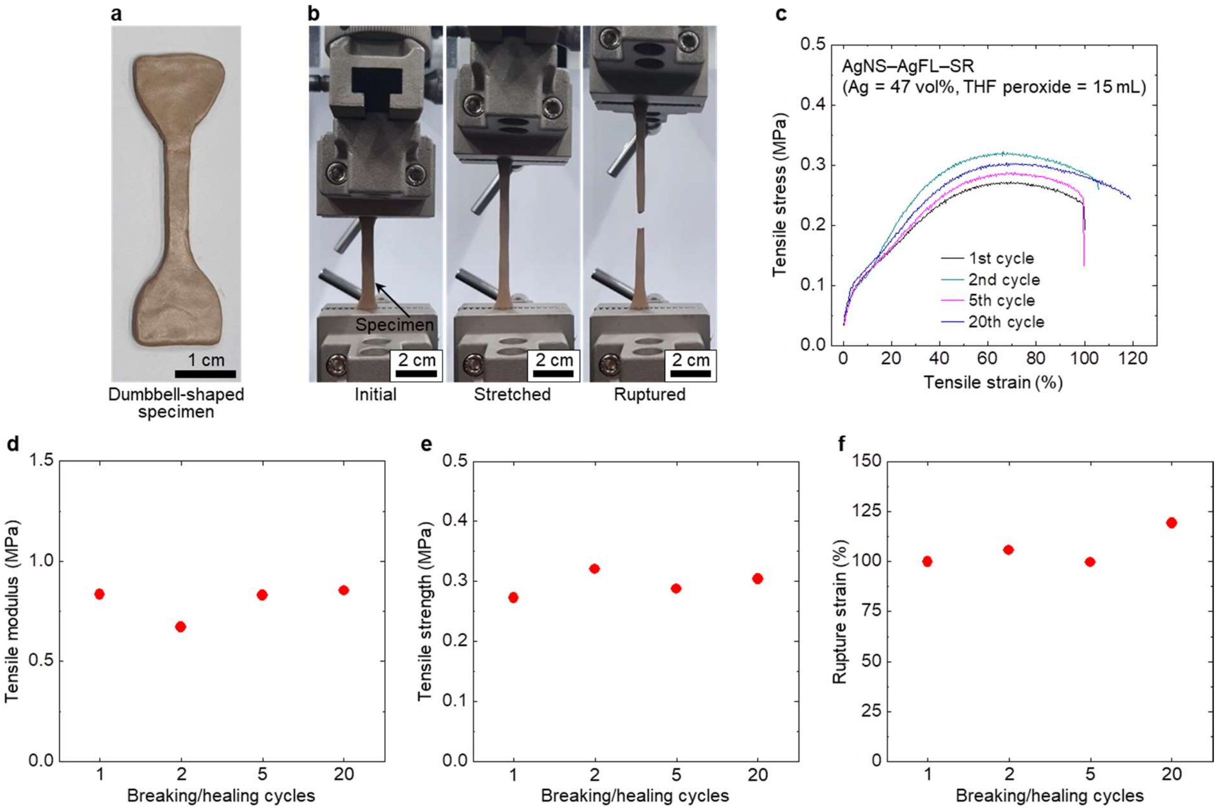

Supplementary Figure 20 The tensile stress-strain characteristics. a An optical image of the dumbbell-shaped AgNS-AgFL-SR (Ag = 47 vol%, THF peroxide = 15 mL) nanocomposite specimen. The freshly synthesized nanocomposite went through a number of pre-breaking/healing cycles to evaporate possibly remnant solvent inside the nanocomposite. The dumbbell-shaped specimen was then manually formed using a polylactic acid mold. The mold was fabricated by a 3-dimensional printer following the ASTM D412-16 standard for tensile tests. **b** The dumbbell-shaped specimen was clamped, stretched, bifurcated, healed, and reshaped for each tensile stress-strain experiment. The fracture occurred at the central test section of the specimen (strain rate = 450 mm min^{-1}). **c** The tensile stress-strain curves of the AgNS-AgFL-SR (Ag = 47 vol%, THF peroxide = 15 mL) nanocomposite during 20 breaking/healing cycles. **d-f** Tensile modulus, tensile strength, and rupture strain.

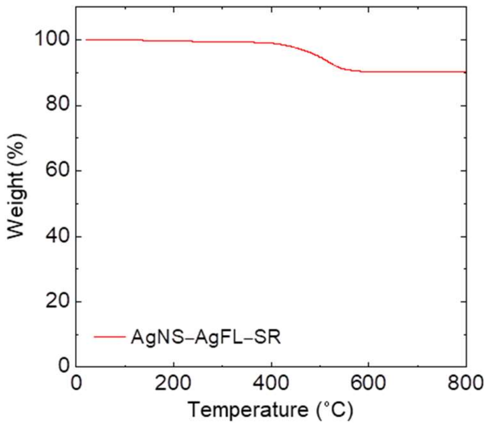

Supplementary Figure 21 Thermogravimetric analysis of the AgNS-AgFL-SR nanocomposite. The total Ag concentration was 44 vol% (THF peroxide = 15 mL).

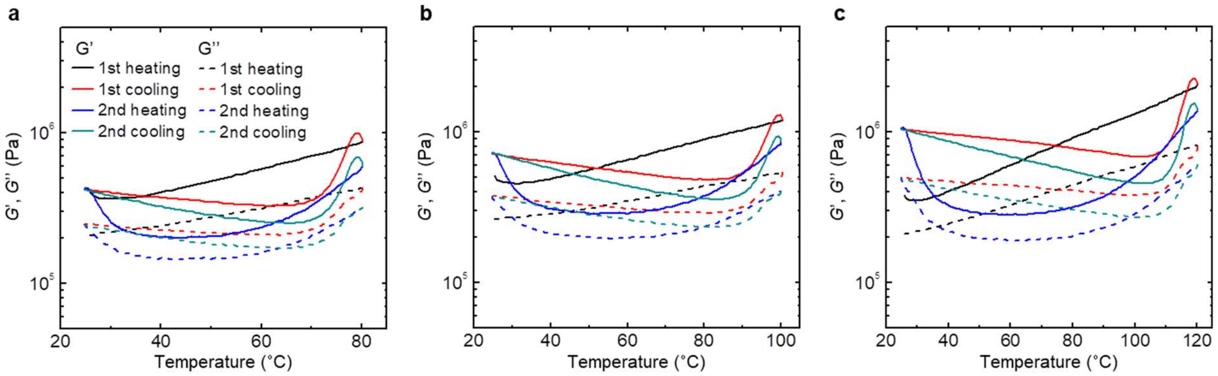

Supplementary Figure 22 The cyclic temperature-sweep rheology measurement. a-c The AgNS-AgFL-SR (Ag = 44 vol%, THF peroxide = 15 mL) nanocomposite (shear strain frequency = 6.28 rad s^{-1}, strain amplitude = 0.03 %, 5 °C min^{-1}) was investigated. Maximum temperatures were 80, 100, and 120 °C, respectively.

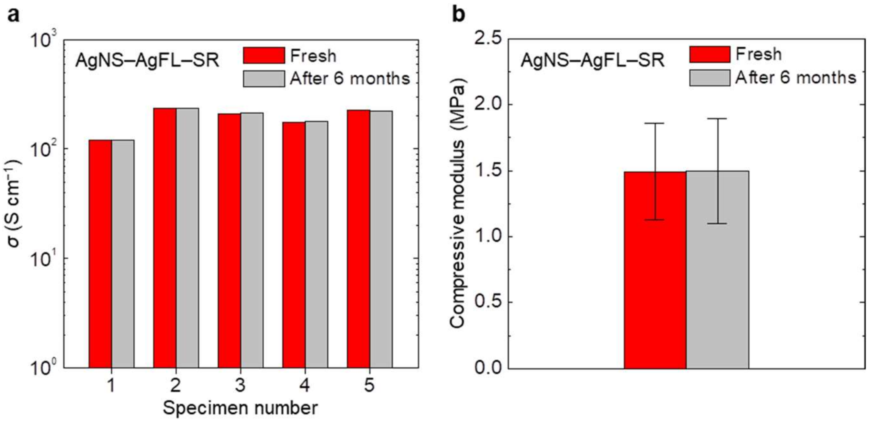

Supplementary Figure 23 Long-term stability analysis. a Electrical conductivity. The AgNS-AgFL-SR (Ag = 44 vol%, THF peroxide = 15 mL) nanocomposite specimens were stored for 6 months in an ambient air environment. **b** Compressive modulus. The error bars represent the standard deviation of the data.

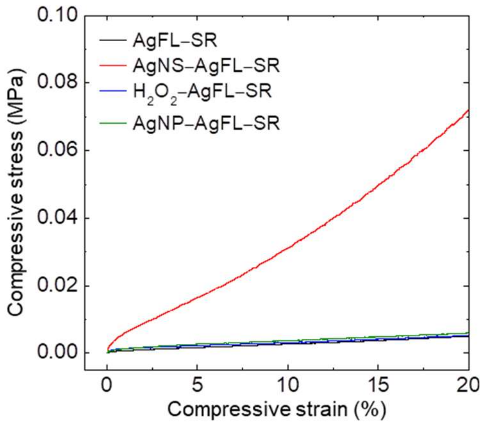

Supplementary Figure 24 The compressive stress-strain analysis. The AgNS-AgFL-SR and control nanocomposites (Ag = 44 vol%) were investigated. The *E* was estimated from the slope of initial linear region of the stress-strain curves.

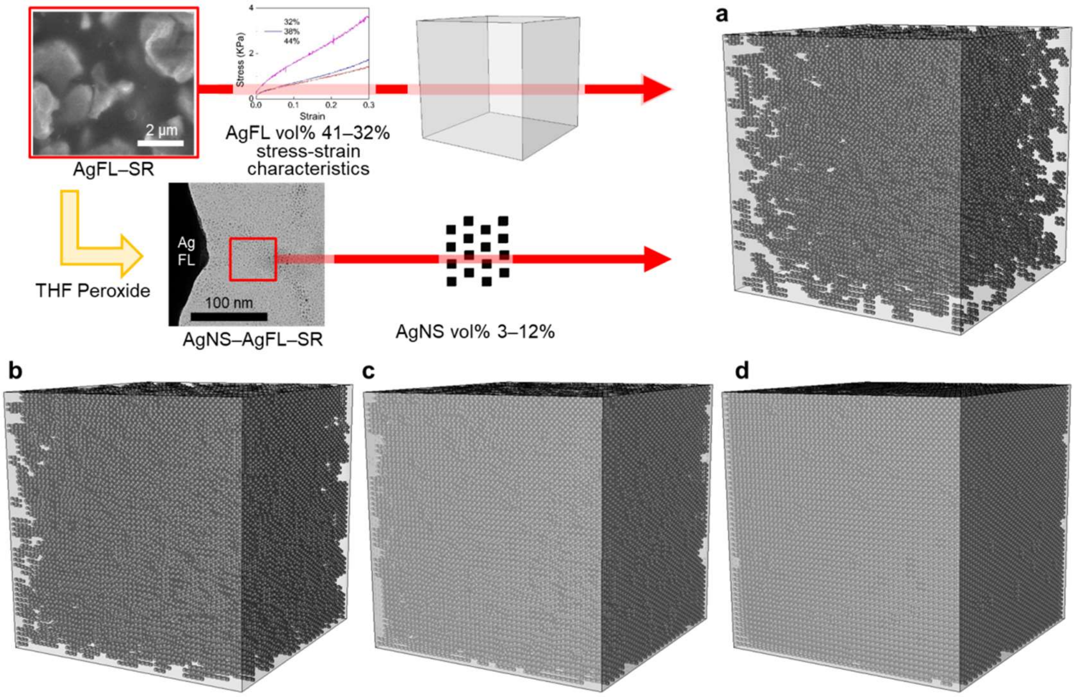

Supplementary Figure 25 Numerical models of the AgNS-AgFL-SR nanocomposites. Each model is obtained by including a definite volume fraction of AgNS particles in the AgFL-SR reference matrix. The total Ag filler fraction was fixed at 44 vol%. The volume fractions of AgNS particles are **a** 3, **b** 6, **c** 9, and **d** 12%, respectively.

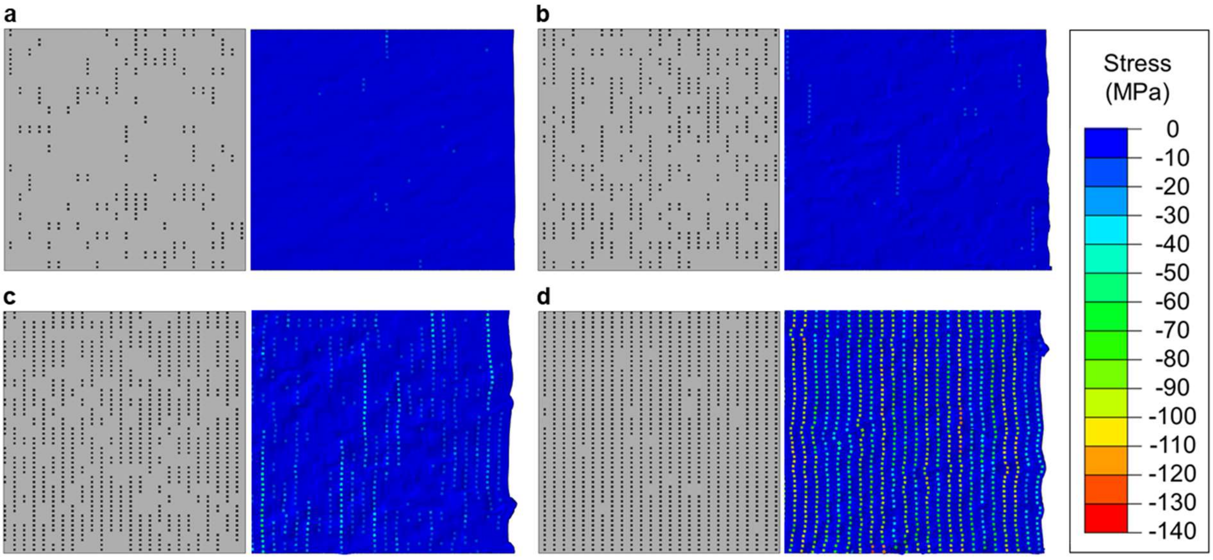

Supplementary Figure 26 The compressive stress distribution at 1% strain. The effect of AgNS particles on the stress of the AgNS-AgFL-SR nanocomposite is shown. All images were captured at the central cross-section of the cube. The vertical displacement of the lower surface was fixed to be zero. Grey and blue images represent the cross-section model and corresponding stress distribution. The total Ag filler fraction was fixed at 44 vol%. The volume fractions of AgNS particles are **a** 3, **b** 6, **c** 9, and **d** 12%, respectively.

Supplementary Table 1 The electrical conductivity and healing efficiency of healable nanocomposites in literature. The nanocomposites were either rigid, flexible, or stretchable at room temperature.

Filler	Polymer matrix	Filler fraction [vol% or wt%]	Electrical conductivity [S cm^{-1}]	Electrical healing efficiency [%]	Maximum breaking /healing cycles	Rigid, Flexible, or Stretchable	Ref
Ag nanoparticle	Polyimine	50 wt%	N/A (Resistance only)	88% (after 1st cycle)<br>63% (after 2nd cycle)<br>53% (after 3rd cycle)	3	Flexible	4
Ag flake	Curcumin polymer	41 vol%	46	N/A	1	Stretchable	5
Graphene	PDMS-COO-Zn	25 wt%	850	N/A	N/A	Rigid	6
Ag flake -Ag nanoparticle	Poly(dimethylsiloxane)-4,4′-methylenebis(phenyl urea)– isophorone bisurea	83 wt%	833	N/A	N/A	Stretchable	7
Ag nanowire	MDPB (2 Maleimide)–FGEEDR (4 Furan) copolymer	N/A	N/A (Resistance only)	97% (after 1st cycle)<br>81% (after 2nd cycle)<br>17% (after 3rd cycle)<br>15% (after 4th cycle)	4	Flexible	8
Ag nanowire	MDPB (2 Maleimide)–FGEEDR (4 Furan) copolymer	N/A	N/A (Resistance only)	89%	1	Flexible	9
Carbon nanotube (CNT), Ag nanowire	DM-80–diethylenetriamine	N/A	N/A (Resistance only)	92% (Aligned CNT sheet)<br>36% (CNT network)<br>34% (Ag nanowire network)	1	Flexible	10
Carbon nanotube	Poly(dimethylsiloxane)-4,4′-methylenebis(phenyl urea)– isophorone bisurea	N/A	N/A (Resistance only)	85%	10	Stretchable	11
GaIn liquid metal alloy	Silicone elastomer	50 vol%	1370	N/A	N/A	Stretchable	12

Supplementary Table 2 The electrical conductivity of moldable viscoelastic nanocomposites in literature that showed putty- or playdough-like behavior at room temperature. The data of the AgNS–AgFL–SR (Ag = 44 and 47 vol%, THF peroxide = 15 mL) nanocomposite are also provided for comparison.

Filler	Polymer matrix	Filler fraction [vol% or wt%]	Electrical conductivity [S cm^{-1}]	Electrical healing efficiency [%]	Maximum breaking /healing cycles	Ref
N/A	Surfactant-containing PEDOT:PSS	N/A	78	N/A	5	13
Reduced graphene oxide	Polyborosiloxane	0.5 wt%	0.9	> 90	6–8	14
Graphite	Branched polyethylenimine	48 vol%	1.98	98% (after 1st cycle)<br>97% (after 5th cycle)	5	15
Graphene	Silicone polymer	15 vol%	0.001	N/A	N/A	16
Multiwalled carbon nanotube	Polyborosiloxane	13.3 wt%	1.21	98%	6	17
Multiwalled carbon nanotube	Modified polydimethylsiloxane	25 wt%	N/A (Resistance only)	> 97 (after 50th cycle)	50	18
Multiwalled carbon nanotube	Hydroxyl silicone oil-boric acid	2 wt%	0.0016	N/A	1	19
Multiwalled carbon nanotube	Hydroxyl-terminated poly(dimethylsiloxane)	9.46 vol%	1.17	76% (after 7th cycle)	7	20
AgNS -Ag flake	Silicone rubber	44 vol% (88.5 wt%)<br>47 vol% (89.7 wt%)	206<br>1020	100% (during 1000 cycles)	1000	This work

Supplementary Table 3 Electrical conductivity of commercially available conductive playdoughs.

Company	Electrolyte	Matrix	Electrical conductivity [S cm^{-1}]
Sciencezone	KCl	N/A	$\leq 10^{-1}$
Squishy circuits	NaCl	N/A	$\leq 10^{-1}$

Supplementary Table 4 Acronyms of specimens used in this study.

Category	Specimen description	Acronyms
Solvent	Tetrahydrofuran with the inhibitor	THF
	Peroxidized tetrahydrofuran<br>(Tetrahydrofuran without the inhibitor stored in air)	THF peroxide
Filler	Pure silver flake	AgFL
	THF-treated Ag flake	THF-AgFL
	Silver nanosatellite particles generated by the in-situ etching and reduction reaction of Ag flakes by THF peroxide	AgNS
	THF peroxide-treated Ag flake	AgNS-AgFL
	Hydrogen peroxide-treated Ag flake	H_2O_2-AgFL
	Hierarchically structured nanoflower-like silver particle	AgNF
Polymer matrix	Pure silicone rubber	SR
	THF-treated SR	THF-SR
	THF peroxide-treated SR	THF peroxide-SR
Polymer matrix nanocomposite	Ag flakes mixed with SR using THF	AgFL-SR
	Ag flakes mixed with SR using THF peroxide	AgNS-AgFL-SR
	H_2O_2-treated Ag flakes mixed with SR using THF	H_2O_2-AgFL-SR
	Commercial silver nanoparticles and Ag flakes mixed with SR using THF	AgNP-AgFL-SR
	AgNFs mixed with SR using THF	AgNF-SR

Supplementary Note 1 Iodometric titration of THF peroxide

The iodometric titration of THF peroxide was carried out using a modified previously reported protocol [21]. Firstly, the analyte solution (THF peroxide, 3 mL) was reacted with excessive amounts of KI (Sigma-Aldrich, 746428, 0.1204 M, 15 mL), H_2SO_4 (Sigma-Aldrich, 339741, 3.5 M, 10 mL), and ammonium molybdate (2 drops). Supplementary Equation 1 shows the stoichiometric reaction formula. The H_2SO_4 facilitated an acidic environment required for the liberation of I_2 while ammonium molybdate acted as a catalyst for the oxidation of THF peroxide [21]. The peroxide moiety in THF peroxide selectively reacted with KI, resulting in the formation of I_2

$$C_4H_8O_3 \text{ (THF peroxide)} + 2KI + H_2SO_4 \xrightarrow{\text{ammonium molybdate}} I_2 + K_2SO_4 + C_4H_8O_2 + H_2O$$

(Supplementary Equation 1)

The color of the analyte mixture solution was deep brown owing to the produced I_2 (Supplementary Figure 2a). $Na_2S_2O_3$ (Sigma-Aldrich, 217263, 0.01M) was then gradually added to the analyte mixture solution using a burette. The color of the analyte mixture solution changed to pale yellow as the iodine was consumed by the oxidation of $Na_2S_2O_3$ (Supplementary Figure 2b and Supplementary Equation 2).

$$I_2 + 2Na_2S_2O_3 \longrightarrow Na_2S_4O_6 + 2NaI \qquad \text{(Supplementary Equation 2)}$$

Starch (Sigma-Aldrich, S9765, 1 mL) was additionally added towards the end of titration to clearly detect the end point of titration (Supplementary Figure 2c). Starch formed a blue-colored starch-iodine complex by reacting with I_2 even at a very low concentration [21]. The titration was continued with $Na_2S_2O_3$ until the solution became colorless (Supplementary Figure 2d). The titration reaction was carried out 3 times, and the average concentration of THF peroxide was 0.068 M (THF peroxide in the initial THF without the inhibitor).

Supplementary Note 2 The NMR analysis of the peroxidation process

The ^{1}H NMR analysis was carried out to measure the concentration of THF peroxide (Supplementary Figure 8a). Dimethyl sulfone was used as an internal standard (0.154 M). The molar concentration of THF peroxide ($C_{\text{THF peroxide}}$) was then calculated using Supplementary Equation 3.

$$C_{\text{THF peroxide}} = \frac{I_{\text{THF peroxide}}}{I_{\text{Dimethyl sulfone}}} \times \frac{N_{\text{Dimethyl sulfone}}}{N_{\text{THF peroxide}}} \times C_{\text{Dimethyl sulfone}} \quad \text{(Supplementary Equation 3)}$$

where I is the integrated peak area of ^{1}H NMR spectrum, N is the number of hydrogen atoms contributing to the peak, and C is the molar concentration. $N_{\text{THF peroxide}}$ and $N_{\text{Dimethyl sulfone}}$ was 1 and 6, respectively. The $C_{\text{THF peroxide}}$ was found to be 0.061 M. This was very close to the concentration measured by the iodometric titration method (Supplementary Figure 2, 0.068 M).

The ^{13}C NMR analysis of THF with BHT inhibitor and THF peroxide was also carried out to confirm the peroxidation of THF in absence of the inhibitor (Supplementary Figure 8b–c). The ^{13}C NMR spectrum of THF with BHT inhibitor exhibited two peaks (Supplementary Figure 8b) [22]. In contrast, the oxidation at the α-carbon atom of THF changed the electronic environment, resulting in four distinct peaks of THF peroxide (Supplementary Figure 8c) [23]. This confirmed the existence of peroxidation.

Supplementary Figure 8d shows the ^{1}H NMR spectrum after the reaction of THF peroxide with AgFLs (AgNS-AgFL). The spectrum of pure THF peroxide before the reaction is also shown for comparison. The residual THF peroxide peak was negligible after the AgNS particle generating reaction (AgNS-AgFL).

Supplementary Note 3 Finite element analysis of the AgNS-AgFL-SR nanocomposite

Finite element analysis (FEA, ABAQUS, SIMULIA) [24] was conducted to simulate the effect of AgNS particles on the mechanical property of the AgNS-AgFL-SR nanocomposite. The AgNS-AgFL-SR nanocomposite was modeled as a unit cube (side length = 0.4 μm). The unit cube was made up of one million C3D8R elements (100 elements each in X, Y, and Z directions), and the size of each element was 4 nm. The size of element was determined considering the average size of small AgNS particles (Fig. 2c). The AgNS-AgFL-SR nanocomposite was considered to be composed of two parts. The AgFLs and SR matrix constituted one part while the AgNS particles comprised the other part. Here after, the first part containing AgFLs and SR was referred to as the reference matrix for clarity. The stress–strain characteristic of the reference matrix was assumed to be the same as that of the AgFL-SR nanocomposite, and the compressive modulus of the AgFL-SR nanocomposite was directly obtained from experiments (Fig. 6a). A definite volume fraction of nanoparticle elements, representing AgNS particles, was introduced into the reference matrix to simulate the mechanical property of the AgNS-AgFL-SR nanocomposite (Ag = 44 vol%). Supplementary Figure 25 shows four AgNS-AgFL-SR nanocomposite models. The AgNS particle volume fractions were 3, 6, 9, and 12 vol%, respectively. The experimentally measured stress-strain data of the AgFL-SR nanocomposites with Ag volume fractions of 41, 38, 35, and 32% (Fig. 6a) were used for the mechanical properties of the reference matrix, respectively. The total Ag filler fraction was fixed at 44 vol% (e.g., AgNS 3 vol% + AgFL 41 vol %) since the AgNS particles were formed by the in-situ etching and reduction reaction of Ag flakes in the SR matrix. The AgNS particles were randomly placed in the cube to theoretically simulate the mechanical properties of the AgNS-AgFL-SR nanocomposite.

Supplementary References

[1] Azizi, S., Namvar, F., Mahdavi, M., Ahmad, M. B. & Mohamad, R. Biosynthesis of silver nanoparticles using brown marine macroalga, Sargassum muticum aqueous extract. *Materials* **6**, 5942–5950 (2013).

[2] Chun, K.-Y. et al. Highly conductive, printable and stretchable composite films of carbon nanotubes and silver. *Nat. Nanotechnol.* **5**, 853–857 (2010).

[3] Smits, F. M. Measurement of sheet resistivities with the four-point probe. *Bell Syst. Tech. J.* **37**, 711–718 (1958).

[4] Zou, Z. et al. Rehealable, fully recyclable, and malleable electronic skin enabled by dynamic covalent thermoset nanocomposite. *Sci. Adv.* **4**, eaaq0508 (2018).

[5] Zhang, Q. et al. An elastic autonomous self-healing capacitive sensor based on a dynamic dual crosslinked chemical system. *Adv. Mater.* **30**, 1801435 (2018).

[6] Lai, J.-C. et al. A rigid and healable polymer cross-linked by weak but abundant Zn(II)-carboxylate interactions. *Nat. Commun.* **9**, 2725 (2018).

[7] Kim, S. H. et al. An ultrastretchable and self-healable nanocomposite conductor enabled by autonomously percolative electrical pathways. *ACS Nano* **13**, 6531–6539 (2019).

[8] Gong, C. et al. A healable, semitransparent silver nanowire-polymer composite conductor. *Adv. Mater.* **25**, 4186–4191 (2013).

[9] Bae, J.-S. et al. The feasibility of healable electronics and mechanical behavior of silver nanowire (AgNW)/healable polymer composite. *Adv. Mater. Technol.* **3**, 1700364 (2018).

[10] Sun, H. et al. Self-healable electrically conducting wires for wearable microelectronics. *Angew. Chem.* **126**, 9680–9685 (2014).

[11] Son, D. et al. An integrated self-healable electronic skin system fabricated via dynamic r econstruction of a nanostructured conducting network. *Nat. Nanotechnol.* **13**, 1057–1065 (2018).

[12] Markvicka, E. J., Bartlett, M. D., Huang, X. & Majidi, C. An autonomously electrically self-healing liquid metal–elastomer composite for robust soft-matter robotics and electronics. *Nat. Mater.* **17**, 618–624 (2018).

[13] Oh, J. Y., Kim, S., Baik, H.-K. & Jeong, U. Conducting polymer dough for deformable electronics. *Adv. Mater.* **28**, 4455–4461 (2016).

[14] D'Elia, E., Barg, S., Ni, N., Rocha, V. G. & Saiz, E. Self-healing graphene-based composites with sensing capabilities. *Adv. Mater.* **27**, 4788–4794 (2015).

[15] Wu, T. & Chen, B. A mechanically and electrically self-healing graphite composite dough for stencil-printable stretchable conductors. *J. Mater. Chem. C* **4**, 4150–4154 (2016).

[16] Boland, C. S. et al. Sensitive electromechanical sensors using viscoelastic graphene-polymer nanocomposites. *Science* **354**, 1257–1260 (2016).

[17] Wu, T. & Chen, B. Synthesis of multiwalled carbon nanotube-reinforced polyborosiloxane nanocomposites with mechanically adaptive and self-healing capabilities for flexible conductors. *ACS Appl. Mater. Interfaces* **8**, 24071–24078 (2016).

[18] Zhong, X., Hu, H. & Fu, H. Self-cleaning, chemically stable, reshapeable, highly conductive nanocomposites for electrical circuits and flexible electronic devices. *ACS Appl. Mater. Interfaces* **10**, 25697–25705 (2018).

[19] Yuan, F. et al. A flexible viscoelastic coupling cable with self-adapted electrical properties and anti-impact performance toward shapeable electronic devices. *J. Mater. Chem. C* **7**, 8412–8422 (2019).

[20] Chen, Y. et al. Shape-adaptive, self-healable triboelectric nanogenerator with enhanced performances by soft solid–solid contact electrification. *ACS Nano* **13**, 8936–8945 (2019).

[21] Jeffery, G. H., Bassett, J., Mendham, J., Denney, R. C. *Vogel's Textbook of Quantitative Chemical Analysis*. 5th edn (Longman Scientific & Technical, UK, 1989).

[22] Wang, Y., Hou, Y. & Song, H. Ring-closing depolymerization of polytetrahydrofuran to produce tetrahydrofuran using heteropolyacid as catalyst. *Polym. Degrad. Stab.* **144**, 17–23 (2017).

[23] Shurvell, H. F. & Southby, M. C. Infrared and raman spectra of tetrahydrofuran hydroperoxide. *Vib. Spectrosc.* **15**, 137–146 (1997).

[24] Lim, J. G. et al. Parametric study for optimal design of an air plasma sprayed thermal barrier coating system with respect to thermal stress. *Surf. Coat. Technol.* **315**, 105–111 (2017).
