# Supplementary material for: Electron tunneling of hierarchically structured silver nanosatellite particles for highly conductive healable nanocomposites
Source: Nat Commun. 2020 May 7;11:2252. doi: 10.1038/s41467-020-15709-8 (PMC7206115; doi:10.1038/s41467-020-15709-8)
Supplement: Supplementary file 2 — Description of Additional Supplementary Files [file 41467_2020_15709_MOESM2_ESM.pdf]

# Description of Additional Supplementary Files

Supplementary Movie 1 :

Emergency electronics repair demonstration by a robot using the conductive healable nanocomposite.
